# Janus Hydrogels: Design, Properties, and Applications

**DOI:** 10.3390/gels11090717

**Published:** 2025-09-08

**Authors:** Wei Guo, Mahta Mirzaei, Lei Nie

**Affiliations:** 1 College of Life Sciences, Xinyang Normal University, Xinyang 464000, China; weiguo@xynu.edu.cn; 2 Department of Environmental Technology, Food Technology, and Molecular Biotechnology, Ghent University Global Campus, Incheon 21985, Republic of Korea; mahta.mirzaei@ghent.ac.kr; 3 Department of Food Technology, Safety and Health, Faculty of Bioscience Engineering, Ghent University, Coupure Links 653, geb. A, B-9000 Ghent, Belgium

**Keywords:** Janus hydrogel, asymmetric property, biomedicine, material science

## Abstract

Janus hydrogels have attracted significant attention in materials science and biomedicine owing to their anisotropic dual-faced architecture. Unlike conventional homogeneous hydrogels, these heterogeneous systems exhibit structural and functional asymmetry, endowing them with remarkable adaptability to dynamic environmental stimuli. Their inherent biocompatibility, biodegradability, and unique “adhesion–antiadhesion” duality have demonstrated exceptional potential in biomedical applications ranging from advanced wound healing and internal tissue adhesion prevention to cardiac tissue regeneration. Furthermore, “hydrophilic–hydrophobic” Janus configurations, synergistically integrated with tunable conductivity and stimuli-responsiveness, showcase the great potential in emerging domains, including wearable biosensing, high-efficiency desalination, and humidity regulation systems. This review systematically examines contemporary synthesis strategies for Janus hydrogels using various technologies, including layer-by-layer, self-assembly, and one-pot methods. We elucidate the properties and applications of Janus hydrogels in biomedicine, environmental engineering, and soft robotics, and we emphasize recent developments in this field while projecting future trajectories and challenges.

## 1. Introduction

Janus hydrogels, named after the two-faced Roman deity Janus [1], represent an emerging class of anisotropic materials engineered with spatially segregated functionalities on opposing surfaces. First conceptualized through Cho and Lee’s seminal work on Janus particles in 1985 [2], these materials have evolved into sophisticated hydrogel systems that integrate asymmetric properties—such as adhesive/antiadhesive interfaces, amphoteric charge distributions, and hydrophilic/hydrophobic domains—within a unified matrix. This intrinsic duality addresses the critical limitations of conventional homogeneous hydrogels, which often exhibit compromised performance when confronted with conflicting operational requirements.

Constrained by structural and functional homogeneity, traditional single-component hydrogels struggle to meet the multifunctional demands of advanced applications. For instance, internal biomedical adhesives require simultaneous robust tissue adhesion and postoperative anti-adhesion capabilities to prevent fibrotic complications [3,4]. At the same time, public disinfection systems require a precise balance between pathogen eradication and avoidance of disinfectant overuse—a challenge exacerbated by environmental persistence and antimicrobial resistance [5]. Janus hydrogels transcend these limitations through the strategic spatial organization of antagonistic properties, enabling concurrent fulfilment of divergent performance criteria.

**Scheme 1:**
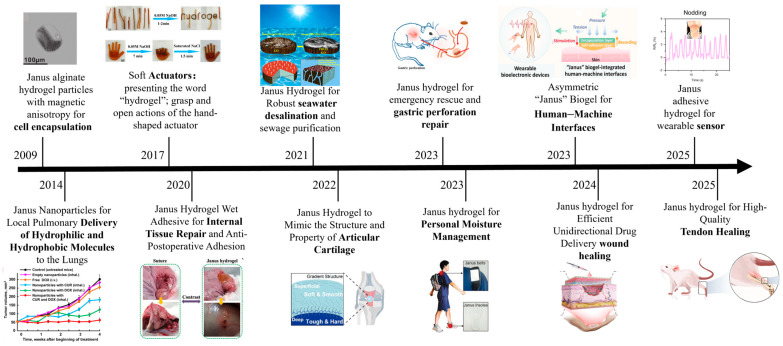
Diagram of hydrogels used in various fields.

Exploring Janus hydrogels has opened new avenues in material science, particularly in biomedical applications and environmental sustainability. Scheme 1 shows the development of Janus hydrogel applications in various fields. By possessing two distinct functionalities on different sides, Janus hydrogels can be engineered to perform complex tasks that traditional, uniform hydrogels cannot achieve. This duality enhances their utility and significantly impacts their design and application. As we delve deeper into the advancements in Janus hydrogels, it is crucial to examine the methodologies employed in their fabrication. Recent studies have highlighted various approaches, including microfluidic techniques, phase separation methods, and photopolymerization [6,7]. Each method offers unique advantages, including precise control over material properties and scalability for industrial applications. For instance, microfluidic techniques enable the generation of Janus hydrogels with nanoscale precision, allowing researchers to fine-tune the surface chemistry for specific interactions [8]. This level of control can lead to the development of hydrogels that selectively interact with specific biological environments, such as tumor tissues, thereby enhancing drug delivery systems [9].

Furthermore, the versatility of Janus hydrogels extends to their potential use in innovative materials. By integrating stimuli-responsive components, these hydrogels can alter their properties in response to environmental triggers, such as pH, temperature, or light [10,11]. This capability is particularly promising for applications in drug delivery, where external stimuli can achieve the controlled release of therapeutic agents, minimize side effects, and increase treatment efficacy. The significance of reviewing the current state of Janus hydrogels lies in their diverse applications and the potential for interdisciplinary collaboration. Materials scientists, biologists, and engineers can collaborate to innovate and push the boundaries of what is possible with these materials. For instance, understanding the biocompatibility and biodegradability of Janus hydrogels is paramount for their application in medical fields. Ongoing research into natural polymer-based Janus hydrogels may lead to breakthroughs in tissue engineering and regenerative medicine, where the integration of these materials can support cell growth and tissue repair. Moreover, as global concerns about environmental sustainability rise, Janus hydrogels can play a crucial role in developing eco-friendly materials. Their potential application in wastewater treatment, where one side of the hydrogel can attract and capture pollutants while the other remains inert, exemplifies their dual functionality in addressing pressing environmental issues [12]. This aspect underscores the importance of further research into the synthesis of Janus hydrogels using renewable resources, ensuring that the materials serve functional purposes and align with sustainability goals.

In conclusion, the advancements in Janus hydrogels represent a significant leap in materials science, with implications that span multiple fields. The unique properties of these hydrogels enable innovative solutions to complex problems in medicine and environmental science. As research progresses, it is essential to continue exploring their potential through comprehensive reviews, fostering collaboration among disciplines, and prioritizing sustainability in material development. The journey of Janus hydrogels is far from over, and their future holds promise for addressing some of the most pressing challenges of our time. While existing reviews on Janus hydrogels predominantly emphasize advancements in biomedical applications, others have examined their utility within specialized domains such as postoperative anti-adhesion barriers or battery devices [13,14,15,16,17]. To the best of our knowledge, no comprehensive analysis systematically integrates the multidisciplinary progress of Janus hydrogels across biomedicine, environmental engineering, soft robotics, sensing technologies, and personal humidity management. This review enriches this content by covering aspects such as design, property, and cross-disciplinary applications. Table 1 exhibits part of the preparation, properties, and applications of Janus hydrogels.

## 2. Types of Janus Hydrogels

Janus hydrogels are a unique class of materials characterized by their anisotropic structures, which exhibit distinct properties on different sides or layers. These hydrogels are broadly categorized into two main types: double-layer Janus hydrogels and multilayer Janus hydrogels. The classification is based on the complexity and arrangement of their structural layers. In this section, we will introduce these two types of Janus hydrogels.

### 2.1. Double Layer

**Figure 1:**
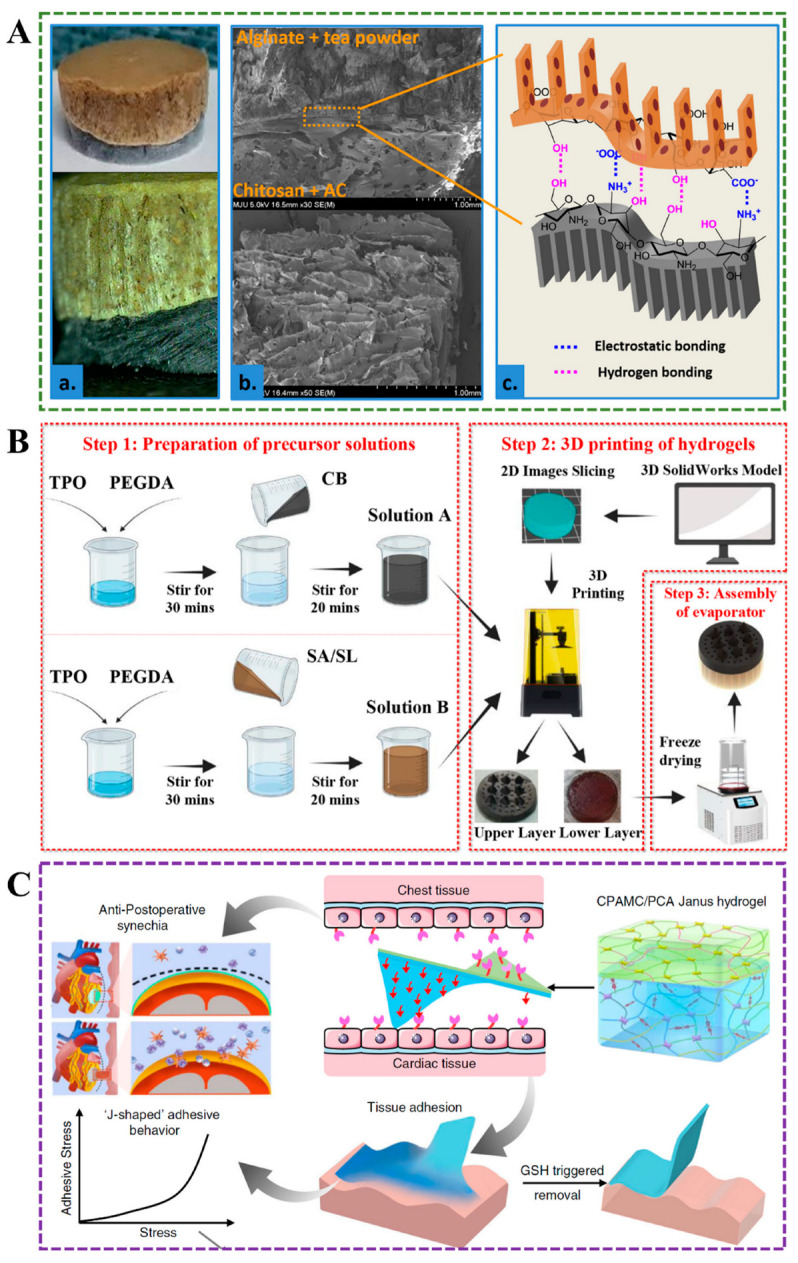
(**A**) The bi-layer Janus hydrogel is composed of a chitosan/activated carbon layer and an alginate/tea powder layer [37]. (**B**) Diagram of preparation of double-layer Janus hydrogel. TPO: diphenyl (2,4,6-trimethylbenzoyl)phosphine oxide [38]. (**C**) Structure and application of the CPAMC/PCA double-layer hydrogel [39].

Double-layer Janus hydrogels, characterized by two distinct layers with tailored chemical, physical, or mechanical properties, have emerged as versatile platforms for multifunctional applications. Their structural simplicity enables precise control over layer-specific functionalities while maintaining fabrication feasibility. Recent advances highlight diverse fabrication approaches and performance optimizations. For instance, Kai Ren et al. developed a PSAP/DXP@AgNPs double-layer hydrogel, where the Poly(*N, N*-dimethylacrylamideco-levodopa acrylamide)/XG/polyethylene glycol diacrylate polymer/silver nanoparticles (DXP@AgNPs) layer exhibits skin-adhesive properties for localized silver nanoparticle delivery, achieving effective bacterial eradication, while the polyethylene glycol diacrylate /sulfobetaine methacrylamide)/*N,N*-methylene bisacrylamide polymer (PSAP) layer prevents bacterial adhesion through its anti-fouling surface [36]. Despite its promising antibacterial efficacy, concerns persist regarding the long-term biocompatibility of AgNPs, particularly their potential cytotoxicity during prolonged applications. In contrast, Abebe et al. proposed an eco-friendly double alginate-chitosan Janus hydrogel tea bag, leveraging electrostatic interactions and hydrogen bonding between the sodium alginate/tea powder layer and chitosan/activated carbon layer (Figure 1A) [37]. This design addresses safety concerns in the food industry by utilizing natural polymers with inherent biocompatibility; however, the scalability and cost-effectiveness of the synthesis method remain underexplored. Three-dimensional printing technology has further expanded the design possibilities for Janus hydrogels. A double-layer poly(ethylene glycol) diacrylate (PEGDA)-based evaporator exemplifies this innovation: the upper carbon black-incorporated PEGDA layer enhances light absorption, while the lower layer containing sodium lignosulfonate (SL) and alginate (SA) promotes water evaporation (Figure 1B) [38]. Critical to this approach is the precise optimization of carbon black (CB) concentration and printing parameters to ensure structural asymmetry and functional performance. In biomedical engineering, He et al. engineered a reversibly adhesive Janus hydrogel cardiac patch composed of a bottom CPAMC layer ((CNC-CHO/PEI/AA)/MASEP/BCA/CA) and a top PCA layer (PEGDA/CNC-CHO/AA) (Figure 1C) [39]. The multifunctional and asymmetric CPAMC/PCA hydrogel is composed of two layers, and the adhesion, electrostatic adsorption, dynamic crosslinking, and permanent crosslinking enable the two layers to interact closely. The bottom CPAMC hydrogel contained acrylic acid (AA), polyethyleneimine (PEI), and aldehyde cellulose (CNC-CHO); the methyl acrylic acid potassium (MASEP) and caffeic acid (CA) were introduced to enhance the adhesion onto tissue, and the N, N’-bis(acryloyl) cystamine (BAC) as dynamic crosslinking agent, which exhibited strain-stiffening behavior for adapting for biological tissue and on-demand removal. The top PCA hydrogel contained AA and carboxylated cellulose (CNC-COOH), polyethylene glycol diacrylate (PEGDA), which reduced the inflammatory response due to the anti-cell-adhesion ability and non-fouling properties [39]. Despite its multifunctionality, the hydrogel’s long-term mechanical stability and reversible adhesion under physiological conditions require rigorous validation for clinical translation. Janus hydrogels, synthesized by combining two hydrogels with distinct properties, are often praised for their bifacial characteristics and ease of synthesis. However, the practical applications and long-term stability of these materials warrant closer scrutiny.

### 2.2. Multilayers

**Figure 2:**
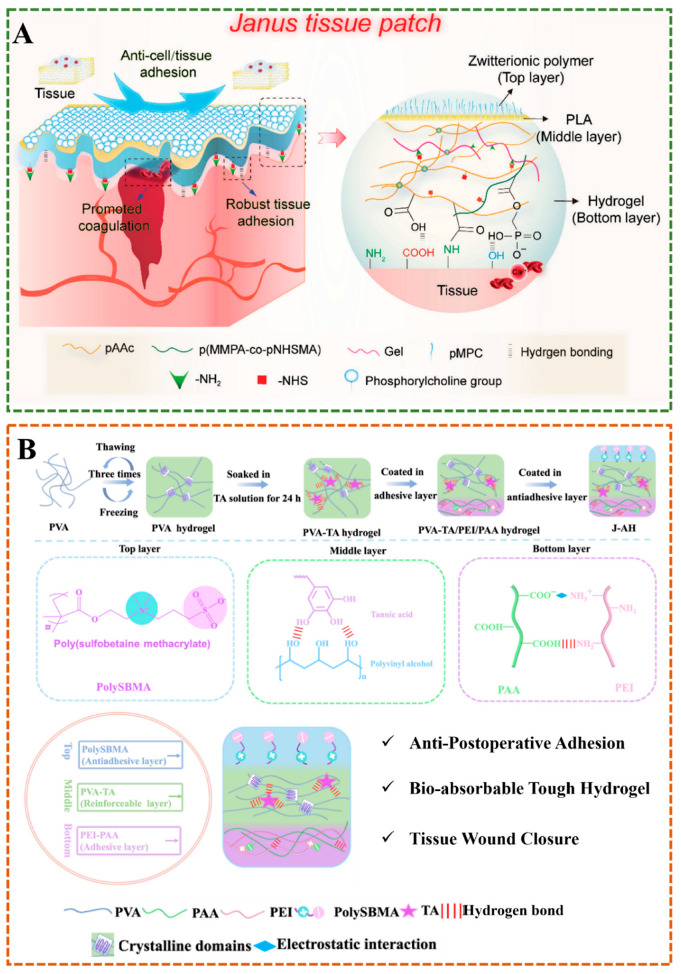
(**A**) The multilayer Janus hydrogel consists of a zwitterionic polymer coating, a PLA skeleton, and a hydrogel substrate for postoperative anti-adhesion [40]. (**B**) Diagram of the synthetic strategy for the three layers of Janus hydrogel [32].

Multilayer Janus hydrogels, composed of more than two layers, offer enhanced functionality and versatility. Their stratified architectures enable precise control over gradient properties (for example, stiffness, porosity, or chemical composition), making them ideal for applications requiring biomimetic interfaces or multifunctional integration. Peng et al. prepared three layers of tissue patches with asymmetric adhesion properties, the bottom layer is crosslinked by methacrylate gelatin with poly(acrylic acid)-co-poly(methacrylic acid N-hydroxysuccinimine) (p(AA-co-NHSMA)) polymer through hydrogen bonding, which can firmly adhere to the bleeding tissue and promote coagulation, the thin polylactic acid as the middle layer can increase the tensile strength of the patch by 132%, and the top layer is zwitterionic polymer coating as an anti-fouling layer to prevent postoperative tissue adhesion (Figure 2A) [40]. Similarly, Lin et al. developed a tri-layered hydrogel system comprising: (1) a zwitterionic anti-adhesion surface, (2) a poly(vinyl alcohol)/tannic acid(PVA-TA) matrix for mechanical reinforcement, and (3) a poly(acrylic acid)/polyethylenimine (PEI-PAA) adhesive base (Figure 2B) [32]. This hierarchical design surpasses the mechanical performance of most conventional hydrogels, highlighting the synergy between layer-specific functionalities. Mao et al. developed a multilayer electrospun Janus nanomaterial using gelatin methacrylate (GelMA), polylactide (PLA), polyglycolide (PGA), and lecithin, with the inner layer of PLA/PGA/Lec, which has excellent mechanical properties and anti-fibroblastic cell adhesion, PLA/PGA/Lec and GelMA mixed nanofibers as the middle layer, and the outer layer of GelMA, which has lubricating properties and acid neutralizing effect, which is expected to be a physical barrier to prevent postoperative adhesion in the abdomen [41]. Miao et al. prepared a three-layer fiber film by electrospinning technology and alkali treatment, consisting of a superhydrophilic outer layer hydrolyzed polyacrylonitrile-SiO_2_ (HPAN), a hydrophobic inner layer of polyurethane (PU), and a transfer layer hydrolyzed PU-PAN (PU-HPAN) in the middle, which can be used as a good functional moisture absorption textile [42]. The additional layers can be engineered to provide gradient properties, such as gradual changes in stiffness, porosity, or chemical composition. This makes multilayer Janus hydrogels ideal for more complex applications, such as mimicking the stratified structure of natural tissues or creating advanced sensors and actuators. The increased complexity, however, also demands more sophisticated fabrication techniques to ensure precise control over the properties and interactions of each layer.

## 3. Design of Janus Hydrogels

Janus hydrogels, renowned for their anisotropic structures and multifunctionality, are designed using various methods, each offering unique advantages in fabrication, control, and application. These methods can be broadly classified into several categories: layer-by-layer, one-pot, self-assembly, electrospinning, and external factor-induced techniques. Each design method offers distinct advantages, allowing researchers to tailor Janus hydrogels for specific applications in biomedicine, engineering, and beyond. In this section, we mainly introduce the design method of hydrogels.

### 3.1. Layer-by-Layer Method

**Figure 3:**
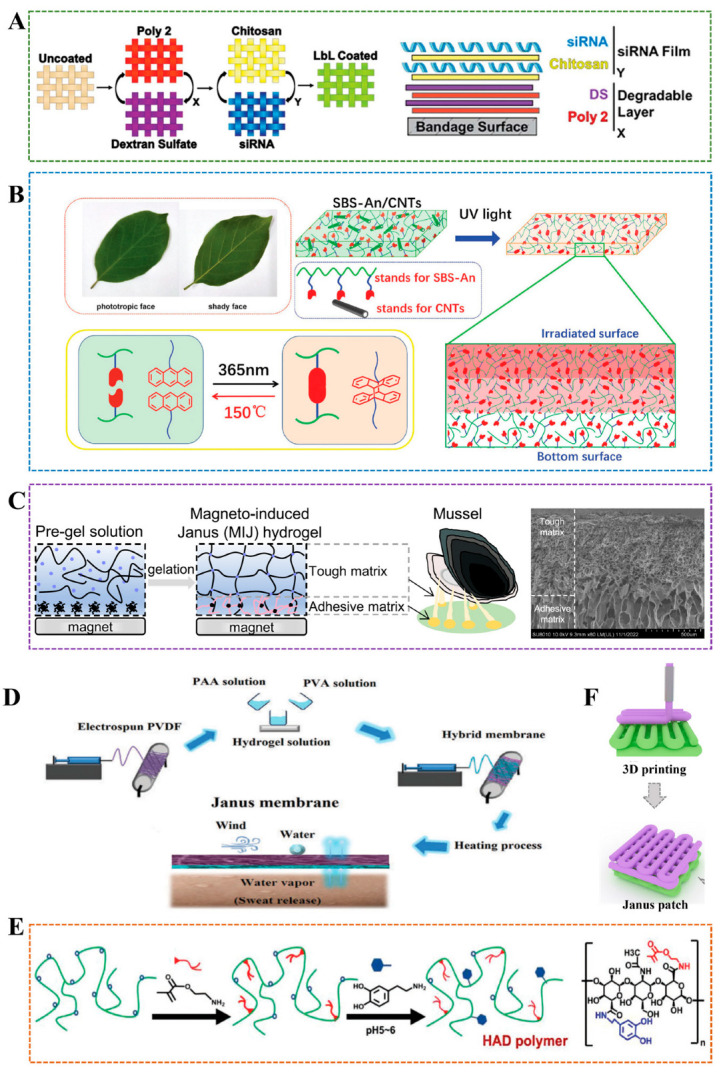
(**A**) Construct ultra-thin siRNA coating by LBL technology [43]. (**B**) The schematic diagram of Janus hydrogel fabricated with anthracene-grafted SBS and CNTs [63]. (**C**) Left: Magneto-induced Janus hydrogel. Right: SEM image of Magneto-induced Janus hydrogel. Scale bar: 500μm [60]. (**D**) The waterproof and breathable Janus membrane was prepared by electrospinning technology [55]. (**E**) Janus hydrogel was prepared by photocrosslinking under ultraviolet irradiation and applied to prevent adhesion formation after operation [64]. (**F**) Janus hydrogel is developed using 3D printing [66].

The Layer-by-Layer (LBL) method involves sequentially depositing different layers of materials to create a Janus structure. This approach enables precise control over the thickness and composition of each layer, making it suitable for applications that require well-defined interfaces, such as drug delivery systems or tissue engineering scaffolds. Castleberry et al. utilized LBL technology to combine self-assembled nanoscale coatings with RNAi, thereby protecting siRNA from degradation and continuously releasing it directly into the wound, thereby achieving rapid healing of chronic wounds [43]. LBL membrane consists of two-layered membrane structures: the first layer is dextran sulfate (DS) and poly(β-amino ester) 2 (poly2), which is used to control the overall degradation rate, and the top layer is chitosan containing siRNA for loading and releasing siRNA (Figure 3A). The membrane architecture can be adjusted by independently changing the number of structural layers of each component to achieve different levels of siRNA release and gene knockout. This method is highly tunable and suitable for various substrates, protecting bioactive molecules, enabling local delivery, and reducing systemic toxicity. However, the synthesis process requires multiple dipping and washing steps, making it complex. Chen et al. uniformly coated a carboxymethyl chitosan/silver (CMCS-Ag) gel layer on a poly(caprolactone)/poly(caprolactone)-poly(citric acid)-co-e-polylysine (PCL/PCL-PCE) nanoscaffold prepared by microfluidic-blow spinning (MBS) technology, and developed a gradient hydrophilic hydrophobic hydrophilic gel Janus nanofiber scaffold using LBL method for wound healing [44]. By adjusting the hydrogel’s fiber structure and pore size, the 3-layer extracellular matrix structure (epidermis, dermis, subcutaneous tissue) of human skin can be simulated. This method also has strong controllability and high functional integration. However, the process is relatively complex, which increases the difficulty and cost of preparation. The interface bonding stability between each layer needs to be optimized.

### 3.2. One-Pot Method

**Figure 4:**
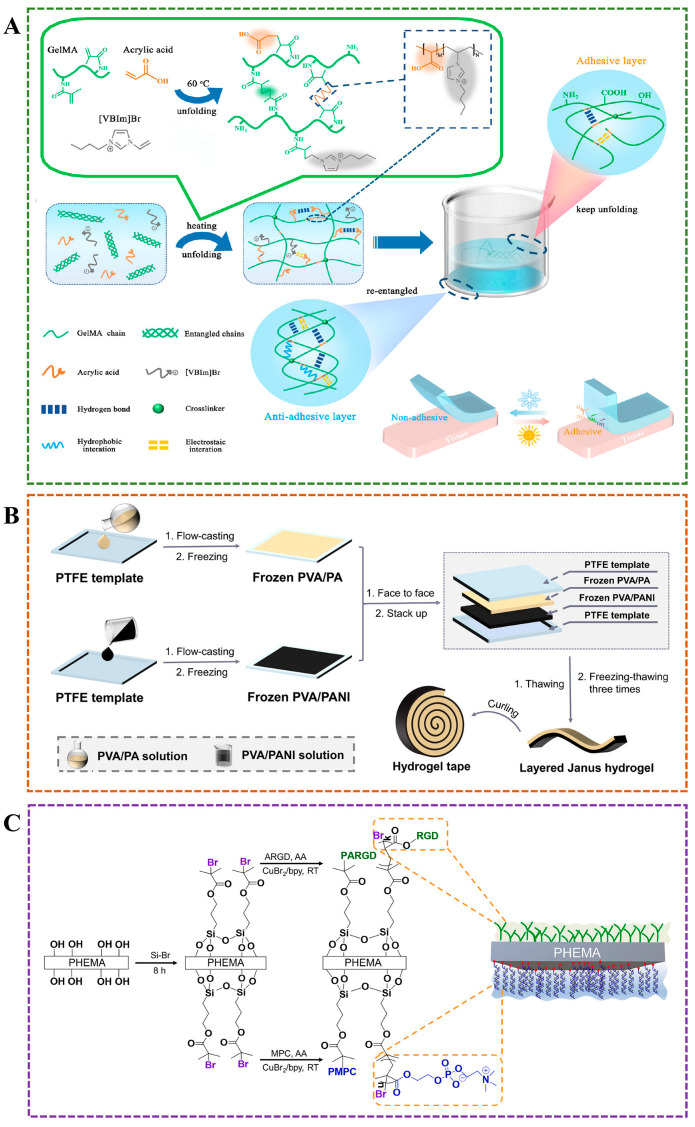
(**A**) Janus hydrogel with asymmetric adhesion prepared by one-pot method for smart adhesion [47]. (**B**) Layered Janus hydrogel with single-sided adhesiveness prepared by cyclic freezing-thawing approach [73]. (**C**) Janus hydrogel with bioadhesive and antifouling properties was constructed using a surface grafting strategy [74].

The one-pot method, in contrast, simplifies the fabrication process by forming Janus hydrogels in a single step, often through phase separation or polymerization. This method is efficient and scalable, but may offer less control over layer boundaries compared to LBL. There are also methods, such as the stepwise synthesis or casting method, which can prepare Janus hydrogels with different adhesion properties. However, these methods involve multiple steps and complex operations, and thus cannot meet the requirements of simplicity, efficiency, economy, and environmental friendliness [45]. In a recent study, PAM-co-LAS hydrogels formed from sodium a-linoleate (LAS) micelles and acrylamide (AM) were synthesized in one step by free radical polymerization using the water-air interface adhesion phenomenon, and the Janus hydrogel exhibited single-sided adhesion due to the influence of surface tension and evaporation effect [45]. Chen et al. combined low-cost and easily processable thermoplastic materials with two-dimensional nanomaterials possessing photothermal properties to develop a smart Janus film that can be obtained in a single step and is expected to be applied in various wearable fields [46]. This strategy is simple and can reduce costs, and it has application prospects in large-scale production. Tang et al. synthesized a Janus supramolecular hydrogel from methacrylate-modified gelatin, acrylic acid (AA), and 1-vinyl-3-butylimidazolium bromide using a simple one-pot method, which exhibited asymmetric adhesion properties due to temperature differences between the skin and cold air and can be separated on demand for wound care (Figure 4A) [47]. The thermoreversible property of the gelatin in this case spontaneously forms an asymmetric structure, a simple process. Fang et al. modified silver ions onto one knob of polystyrene@silica (PS-SiO_2_) particles, and the obtained PS-SiO_2_@Ag Janus particles were mixed with polyacrylic acid and poly-urushiol to form a hierarchical adhesive oil–water Janus hydrogel by a one-pot process, which showed asymmetric interface toughness on the tissue surface and has excellent hemostatic effect [48].

### 3.3. Self-Assembly Method

The self-assembly method utilizes the inherent characteristics of molecules or polymers to form Janus structures autonomously. This approach is particularly beneficial for fabricating intricate, hierarchical architectures with minimal external influence. Amphiphilic molecules possess the ability to self-organize into fibrous configurations. Sami Nummelin and colleagues synthesized amphiphilic Janus dendrimer macromolecules that generated self-assembled hydrogels at a remarkably low mass fraction (0.2 wt%) [49]. By modifying the positioning and quantity of hydrophobic alkyl groups within the Janus dendrimer macromolecules, the mechanical attributes of hydrogels could be fine-tuned, enabling the resultant hydrogels to encapsulate bioactive entities, including peptide proteins and small-molecule pharmaceuticals. In a separate investigation, Tristan Hessberger and co-researchers employed temperature-responsive materials during the synthesis of amphiphilic molecules, producing interfacial self-assembly Janus hydrogels exhibiting dual thermal responsiveness. These hydrogels consisted of a hydrophobic liquid crystalline elastomer on one side and a hydrophilic poly (N-isopropylacrylamide) (pNIPAAm) on the other, capable of transitioning among four distinct particle morphologies as the temperature varied from 20 to 200 °C [50]. Modulating the cross-linking density of NIPAAm enables the adjustment of the amphiphilic properties of Janus hydrogels, facilitating self-assembly at the liquid–liquid interface between aqueous and organic solvents. Interface strategies, such as liquid–liquid and gas–liquid interfaces, are frequently employed to fabricate Janus films [51,52]; however, these methodologies are often complex and challenging to control. In contrast, Luo et al. successfully developed Janus films using a straightforward density deposition technique, eliminating the need for interfaces or sophisticated equipment [53]. This versatile PVA/GO/h-BN Janus film is created by exploiting the density disparity between h-BN and graphene oxide (GO) fillers during solvent evaporation. The resulting film exhibits optical, electrical, and thermal anisotropy, presenting significant potential for applications in optical switching, electronic devices, and thermal management in human systems.

### 3.4. Electrospinning

Electrospinning is another approach that produces fibers with Janus characteristics by applying an electric field to a polymer solution. This method is promising for preparing waterproof and breathable membranes to replace those lacking porous structures and expensive membranes [54]. Amini et al. used poly(vinylidene fluoride)(PVDF), which has the advantages of waterproofing and good thermal stability, electrospun hydrogels as the porous hydrophilic layer of Janus film to improve the permeability of the waterproof film, and prepared a hybrid film with both hydrophilic and hydrophobic properties, which showed good water resistance and permeability (Figure 3D) [55]. The porous structure of Janus hydrogels not only facilitates water resistance and breathability but also facilitates the transport of nutrients and wastes. Zhang et al. used electrospinning to prepare injectable Janus hydrogels with microporous structures, based on the scanning electron microscopy (SEM) observation, these Janus fibers could undergo a sol–gel transformation under thermal induction and hydrophobic action, achieving self-curving at body temperature to form a porous structure to facilitate the transport of nutrients and wastes, which can be applied in tissue engineering [56]. Other types of hydrogels with asymmetric properties can also be developed by electrospinning. For example, Kimna et al. coated the coating material PVA/mucin on the dopamine-conjugated hyaluronic acid polymer by electrospinning to prepare a double-layer hydrogel with asymmetric adhesion properties [57]. Using the asymmetric wettability of lotus leaves, Zhang et al. electrospun a polyacrylonitrile fiber containing PDA onto polypropylene to develop a nanofiber membrane with Janus structure [58]. The membrane promoted the transfer of wound exudate from the hydrophobic side to the hydrophilic side. Moreover, the photothermal effect accelerated the evaporation of the exudate, avoiding frequent dressing changes.

### 3.5. External Factor-Induced Method

The combination of two or more layers is usually unstable, with an obvious bonding problem, resulting in structural damage to Janus and affecting the integrity and function of the hydrogel. This problem can be solved using the method induced by external factors to obtain the gradient structure without an obvious interface [59]. Xu et al. prepared magnetic nanoparticles (Fe_3_O_4_@PDA) coated with polydopamine (PDA) through by self-polymerization of dopamine on the surface of Fe_3_O_4_, the dispersed Fe_3_O_4_@PDA solution into a mold removed the water, and allowed it to accumulate at the bottom under the magnetic field(Figure 3C) [60]. Then, they poured the precursor solution PAAm/Alg-Ca, which was prepared by mixing solution A (acrylamide, N,N′-methylenebis(acrylamide), and calcium sulfate dihydrate), and solution B prepared with sodium alginate, into the mold. Free-radical polymerization was then carried out at 60 °C for 3 h. Under the magnetic field, Fe_3_O_4_@PDA was fixed at the bottom of the mold. Due to the rich catechol groups, which can form an adhesive layer through hydrogen bonds, electrostatic interactions, and coordination bonds, the PAAm/Alg-Ca double network formed the upper resilient matrix. The SEM image in Figure 3C showed no obvious physical interface between the two layers, avoiding the stress mismatch problem in traditional double-layer structures. Moreover, compared to the tough matrix layer, the adhesive layer showed a loose pore structure. This magnetic induction method locates the nanoparticles before polymerization, effectively inhibiting the diffusion of functional molecules in traditional gradient hydrogels over time, and ensuring more stable performance. The external magnetic field-induced method can not only ensure the integrity of the hydrogel’s function but also address many challenges faced by microrobots in the biological field, such as the presence of toxic substances and the limitations of wireless operation. For example, Ali et al. prepared Janus microspheres by mixing sodium alginate containing iron oxides with sodium alginate containing living cells, and obtained a hydrogel robot that could be driven quickly and simply by magnetic fields [61]. Ando et al. formed microdroplets from the pre-gel solutions of the glucose-sensing hemisphere and the pH-sensing hemisphere under the action of centrifugal force and photopolymerized them under ultraviolet irradiation to form a Janus hydrogel microbead fluorescence sensor, which could simultaneously calibrate pH and accurately measure glucose concentration [62]. The development of the Janus composite membrane enables shape transformation under remote control by photothermal stimulation, demonstrating excellent potential for application in the field of intelligent sensors. The anthracene-grafted poly(styrene-*block*-butadiene-*block*-styrene) (SBS) was crosslinked by anthracene dimerization under ultraviolet light. At the same time, the dedimerization was achieved by heat treatment (150 °C), and the introduction of carbon nanotubes controlled the penetration of light in the thickness direction. Janus films with a thickness gradient structure, similar to the principle of leaf photosynthesis, were obtained. These films feature carbon nanotubes with excellent photothermal conversion properties, and the shape change in the membrane can be controlled remotely through the laser region (808 nm) (Figure 3B) [63]. Most asymmetric hydrogels are prepared by interfacial coating or electrospinning, which lack injectable properties and are difficult to use in laparoscopy. Wu et al. synthesized an injectable photocurable Janus hydrogel with hyaluronic acid grafted 2-aminoethyl methacrylate and 3.4-dihydroxy-phenylalanine (HAD), which has broad application prospects in preventing adhesion after minimally invasive surgery(Figure 3E) [64]. The HAD precursor solution first adhered to the tissue and was then irradiated with ultraviolet radiation to achieve the adhesion performance of bearing 50 g. However, the HAD precursor was first UV light-linked to form a hydrogel, which could not adhere to various organs and materials. In another study, the injectable photocurable HAD Janus hydrogel loaded with pluripotent stem cell-derived cardiomyocyte exosomes demonstrated great potential for use in cardiac surgery [65]. External factor-induced methods rely on external stimuli such as temperature, light, or magnetic fields to induce the formation of Janus structures. These methods enable dynamic control over the hydrogel’s properties, making them suitable for responsive or innovative materials.

### 3.6. Others

In addition to the above methods, there are other techniques to prepare Janus hydrogels, such as unilateral dipping, cyclic freeze–thaw, and 3D printing. A combination of chemical and physical crosslinking mechanisms for preparing Janus hydrogel is straightforward and requires no specialized equipment. For example, An et al. placed a layer of non-crosslinked gelatin in a Petri dish and then poured a gelatin/dopamine/nano-clay (GPC) hydrogel solution on top [67]. After one hour of crosslinking at room temperature, a Janus hydrogel was formed. This method utilized the ability of nano-clay to suppress excessive oxidation of dopamine, thereby preserving more catechol groups and enhancing interfacial adhesion. Other strategies involve sealing adhesive groups to mitigate excessive adhesion for constructing Janus hydrogels. For instance, Li et al. fabricated an adhesive hydrogel from polyacrylic acid (PAA) and gelatin (GA) under UV irradiation, and then uniformly coated it with poly(vinyl alcohol) (PVA). Upon drying, a dense physical barrier layer formed, resulting in a PVA/Gel–PAA hydrogel with asymmetric adhesion [68]. SEM images revealed that the adhesive side possessed a porous structure conducive to cell growth, while the non-adhesive PVA coating exhibited a dense, non-porous morphology that effectively resisted protein and cell adhesion. The bonding between the PVA barrier and the Gel–PAA adhesive layer in the PVA/Gel–PAA hydrogel was achieved through hydrogen bonding and mechanical interlocking—a purely physical combination that avoids the use of toxic chemical crosslinkers and may also face the risk of delamination. Inspired by the charge balance characteristics of double-charged ion materials, Peng et al. utilized the charge balance conversion strategy to form distinct charge states in the hydrogel layers of water gels with similar chemical compositions under different pH conditions [69]. They achieved the formation of an adhesive layer rich in -COOH and -NH3^+^ under acidic conditions and an anti-adhesion layer that mimics the overall electrical neutrality of amphoteric ion materials under neutral conditions. Without the need for complex multi-layer designs or chemical modifications, Janus hydrogel can be prepared simply by adjusting the pH to control the charge state. Zhang et al. prepared a multifunctional bandage with asymmetric wettability by coating Polydimethylsiloxane (PDMS) unilaterally on cotton loaded with hydroxylpropyltrimethylammonium chloride chitosan (HACC) by electric spraying method, which can effectively control exudate, prevent wound infection, and promote wound healing [70]. Hu et al. obtained Janus nanosheets of silica with one-side poly(acrylic acid) (PAA) grafting by water-in-oil high internal phase emulsion template method, which can stabilize water-in-water emulsions and is a promising amphiphilic Pickering stabilizer [71]. Xu et al. applied tannic (TA) on one side of the poly [2-(methacryloyloxy)ethyl] dimethyl-(3-sulfopropy)/poly(vinyl alcohol) (PSBMA/PVA) hydrogel by simple unilateral impregnation to obtain a Janus hydrogel with self-adhesive and antifouling properties [72]. Utilizing the advantages of 3D printing, which can be manually controlled, the researchers prepared a double-layer hydrogel with a Janus structure, which can be more effectively applied in the energy and biomedical fields (Figure 3F) [38,66]. Wang et al. proposed a cyclic freeze–thawing method and successfully prepared a Janus hydrogel tape composed of a PVA/phytic acid adhesive layer and a PVA/polyaniline non-adhesive layer, which can be used to prepare functional, flexible sensors with low response time, reversible response, and high sensitivity (Figure 4B) [73]. Han et al. developed a Janus film with asymmetric adhesion properties by grafting Arg-Gly-Asp (RGD) peptide with cell adhesion ability on one side of poly(2-hydroxyethyl methacrylate) (PHEMA) bulk material and an anti-adhesion poly(2-methacryloyloxyethyl phosphorylcholine) (PMPC) on the other side through a surface grafting strategy (Figure 4C) [74]. Table 2 presents a comparison of various methods for preparing Janus hydrogels.

## 4. Desired Properties of JANUS Hydrogels

The accelerated advancement of Janus hydrogels over the past ten years can be attributed primarily to the inadequacy of conventional homogeneous materials in fulfilling the demands of specific application contexts. For instance, numerous biomedical materials require enhanced capabilities to effectively respond to the intricate and dynamic physiological environments beyond the fundamental functionalities of wound adhesion and postoperative tissue protection. The unique property of Janus hydrogels is their two-sided structure, which allows them to exhibit different functions on each side. This dual function not only enables customized responses to various stimuli but also enhances their versatility in applications ranging from drug delivery systems to tissue-engineered stents. In this section, we focus on the different properties of hydrogels.

### 4.1. Efficient Drug Delivery

**Figure 5:**
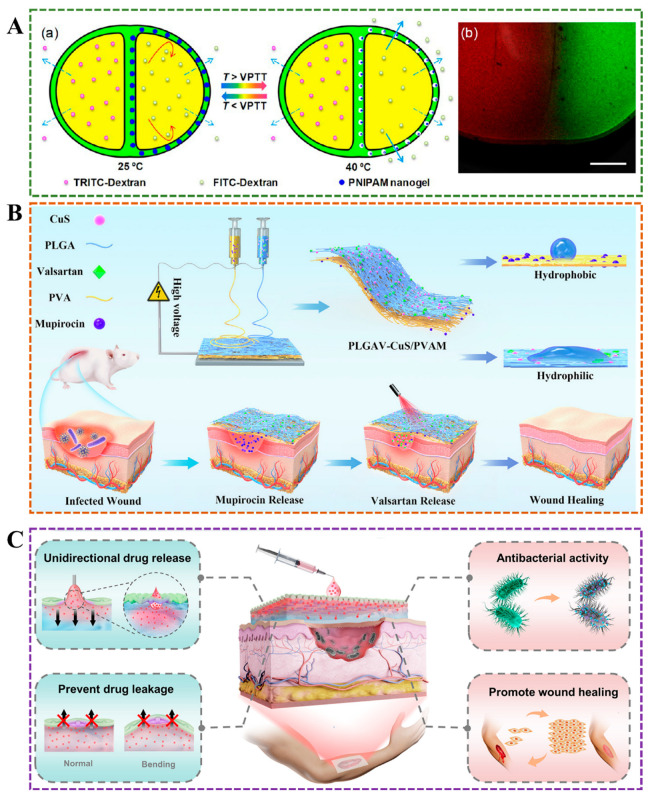
(**A**) In two-compartment capsules, the release rate of TRITC-dextran remained constant in one chamber, and the release rate of FITC-dextran in the other chamber changed with temperature [77]. (**B**) In the early stage, hydrophilic antibacterial drugs (mupirocin) were slowly released in the hydrophilic layer of Janus hydrogel, and, in the later stage, the photothermal effect accelerated the release of anti-inflammatory drugs (valsartan) in the hydrophobic layer, achieving a controllable drug release system during the wound healing process [78]. (**C**) Janus hydrogel delivers the drug from the outside to the wound site in a unidirectional, preventing drug leakage [80].

Janus hydrogel excels in drug delivery due to its unique asymmetric structure, enabling controlled and sustained release. Its dual-faced composition enables tailored interactions with various drugs, thereby enhancing encapsulation efficiency and targeted delivery. This adaptability ensures optimal therapeutic outcomes with minimized side effects. Synovial fluid and tear fluid have special viscoelastic rheological properties. When there is no external pressure, they behave as viscous liquid, and, when pressure is applied, they mainly behave as elastic and prevent friction. Viscoelasticity as the Janus property provides an important guarantee for biological liquids to play their physiological functions; for example, artificial tear liposome drug delivery system can be used to relieve dry eye [75]. The differences in the composition and chemical structure of drugs result in significant variations in their solubility, and cross-contamination between different drugs may also lead to unpredictable consequences. Traditional drug delivery systems are difficult to achieve simultaneous loading of drugs with different solubilities, and cannot avoid cross-contamination between different drugs. Janus particles can load multiple drugs and independently release different drugs due to their asymmetric structure [76]. He et al. developed a multicompartment capsule to achieve a controllable release sequence and rate (Figure 5A) [77]. Shi et al. used polylactic-glycolic acid/copper sulfide nanoparticles and antibacterial agents (valsartan) as hydrophobic layers and poly(vinyl) alcohol and anti-inflammatory agents (mupirocin) as hydrophilic layers to prepare Janus amphiphilic fiber membranes by electrospinning, so as to achieve controllable release of antibacterial agents and anti-inflammatory agents and promote the process of skin wound healing (Figure 5B) [78]. The delivery method for treating chronic wounds typically involves encapsulating drug molecules in wound dressings, such as sponges and medical gauze [79]. However, the efficiency of this traditional delivery method is low, and the secondary injury caused by frequent dressing changes further delays wound healing. Wang et al. prepared a wound dressing with Janus wettability by combining hydrogel formed by chitosan quaternary ammonium salt (HACC), poly(vinyl) alcohol (PVA) and polyacrylic acid (PAA) crosslinked on one side of the hydrophobic finished bandage, which realized unidirectional drug delivery to the wound bed, effectively preventing drug leakage, and high mechanical flexibility ensured the high efficiency of drug delivery while avoiding secondary injuries caused by dressing replacement (Figure 5C) [80].

### 4.2. Biofluid Transport Capability

**Figure 6:**
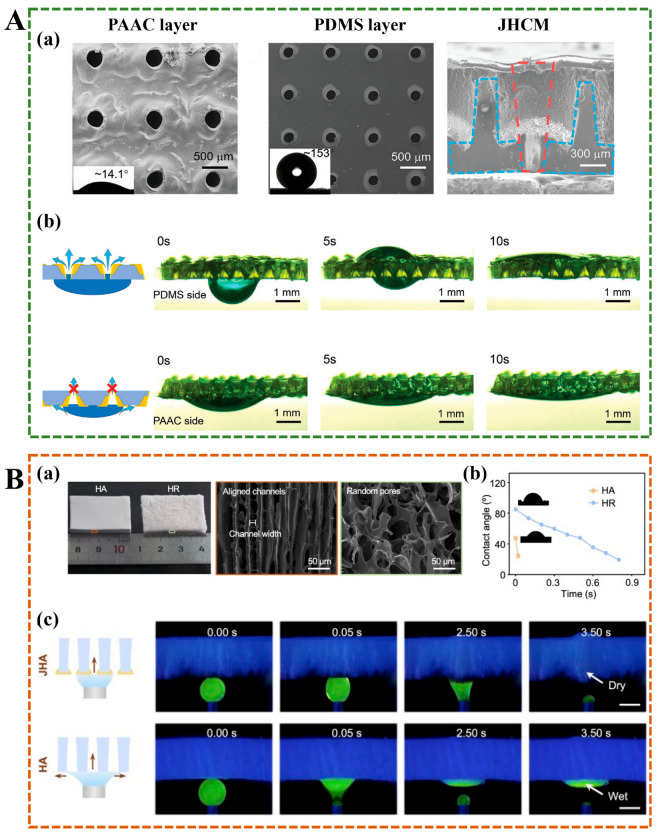
(**A**) (**a**) SEM images of the PAAC layer, the PDMS layer, and the cross-section of JHCM. (**b**) The asymmetric water drop absorption and transport capacity between the two PAAC layer and PDMS layer [82]. (**B**) (**a**) Optical images and SEM images of HA and HR. (**b**) As time increases, the apparent contact angle of water on the HA and HR surfaces. (**c**) The transport of water on the JHA surface and the HA surface. Scale bar, 1 mm. [83]. JHA: Janus hydrogel with aligned channels.

Janus hydrogels exhibit exceptional liquid transport properties attributed to their asymmetric architecture, which establishes distinct hydrophilic and hydrophobic domains. This structural duality facilitates directional fluid migration through wettability gradients, rendering them particularly effective for applications demanding precise liquid control and optimized fluid management. In clinical wound care scenarios, conventional hydrophilic hydrogel dressings often encounter limitations due to their inability to effectively discharge absorbed exudate. The retention of wound fluids impedes continuous drainage, elevates infection risks, and delays the healing process—a critical challenge in chronic wound management. Innovative fabrication strategies have been developed to engineer Janus-structured wound dressings with anisotropic wettability to address this limitation. Zhang et al. employed layered microfluidic spinning technology to construct Janus textiles with spatially differentiated surface properties. The resultant wettability gradient enables the active pumping of wound exudate from the hydrophobic to the hydrophobic surface, effectively maintaining optimal moisture balance while preserving wound breathability [81]. Further advancements in Janus membrane design were achieved through femtosecond laser ablation techniques. Zhang et al. developed a Janus hydrogel composite membrane (JHCM) that integrates hydrophobic polydimethylsiloxane (PDMS) and hydrophilic poly(acrylic acid-co-acrylamide) (PAAC), leveraging the high precision, controllable pore geometry, and tunable pore size afforded by femtosecond laser processing [82]. SEM images revealed that the pore sizes on the PDMS layer are approximately 200 μm, whereas those on the PAAC layer measure about 300 μm (Figure 6A). The smaller pore size on the hydrophobic side compared to the hydrophilic side, combined with the conical pore morphology visible in cross-sectional images, facilitates unidirectional fluid transport from the hydrophobic to the hydrophilic layer. When a water droplet was deposited on the PDMS side, it rapidly penetrated and transferred to the PAAC layer. In contrast, water droplets applied to the PAAC layer spread laterally along the surface without wetting the underlying PDMS layer (Figure 6A). This biofluid transport capability surpasses that of commercial bandages in maintaining wound dryness and cleanliness—an essential feature for diabetic patients prone to excessive wound exudation. The microstructure of hydrogels plays a critical role in governing biological fluid transport. In another study, Xiao et al. designed a self-pumped dressing with arrayed channels, featuring poly(ethylene glycol) diacrylate (PEGDA) as a hydrophilic layer and polyurethane/graphene oxide/polytetrafluoroethylene (PU/GO/PTEE) as a hydrophobic layer (Figure 6B) [83]. SEM images demonstrated that the hydrogel with aligned channels (HA) exhibited a smooth surface and an internally ordered, regular channel architecture. In contrast, the hydrogel with random pores (HR) displayed a rough surface and a disordered, tortuous pore structure (Figure 6B). The aligned microchannels provide strong capillary forces, enabling ultrafast water absorption within 0.02 s and promoting efficient liquid transport from the hydrophobic to the hydrophilic side (Figure 6B) [83].

### 4.3. Mechanical Performance

Hydrogels have high water content, but their mechanical properties are often poor, which limits their application range. The multi-reactivity of Janus nanoparticles (JNs) can effectively improve the mechanical properties of multi-network hydrogels. Hou et al. grafted quaternary ammonium salt on one side of the nanoparticles and an amino group on the spherical side, and the R_3_N^+^-JN-NH_2_ obtained was used as a crosslinking agent to bind with glycol chitosan/sodium alginate double-network hydrogel [84]. Within a certain range, the mechanical properties of the hydrogel became better and better with the increase of R_3_N^+^-JN-NH_2_ content. Chen et al. prepared a hydrogel-Janus nanofiber scaffold (NFS) with superior mechanical properties. The NFS exhibited a high tensile strength of 26.36 ± 1.77 MPa and an impressive fracture elongation of 270 ± 10%, which is comparable to the mechanical properties of human skin (200 ± 15.6%) [44]. The introduction of PCE and CMCS-Ag layers enhanced the stiffness and elasticity of the hydrogel-Janus NFS, as evidenced by the increased tensile strength and elongation compared to pure PCL nanofiber scaffolds. Additionally, the scaffold maintained significant mechanical strength (14.38 MPa) even after immersion in water for 2 h, indicating its robustness in wet environments.

### 4.4. Antibacterial Properties

**Figure 7:**
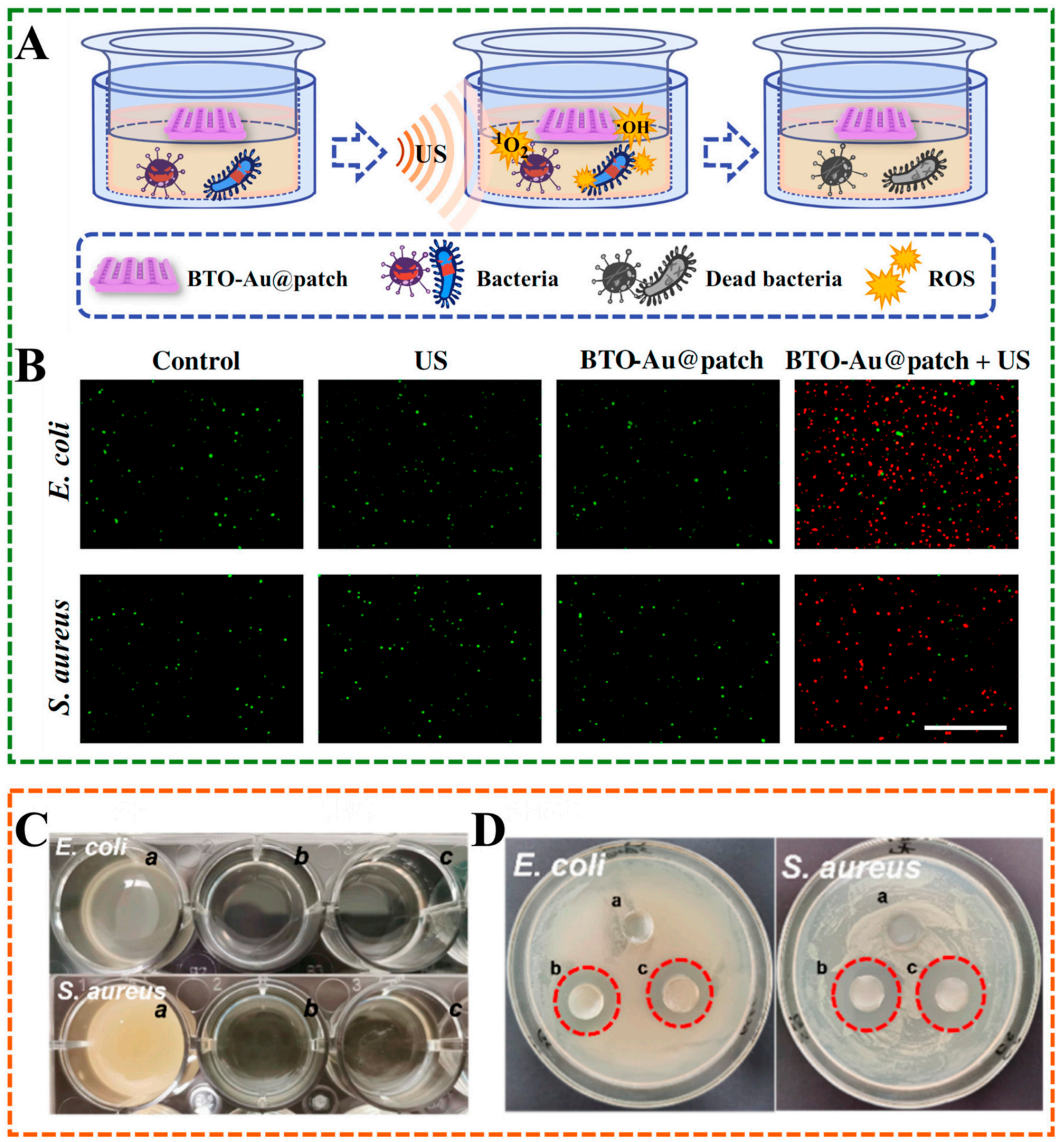
(**A**) Schematic illustration of piezoelectric reactive oxygen species (ROS) generation under ultrasound (US) stimulation and the sonodynamic antibacterial process in Janus patch. (**B**) Enhanced antibacterial performance of the Janus patch under ultrasound-triggered sonodynamic therapy [66]. (**C**) The concentration of bacteria after 24 h with SH, SHG, SHGC hydrogel treatment. (**D**) The bactericidal effect of SH, SHG, SHGC hydrogels (a, b, and c are SH, SHG, and SHGC hydrogels, respectively) [86].

Infection is the leading cause of delayed postoperative repair and wound healing, which brings both physical and mental harm to patients and increases the economic burden. Huang et al. used an extrusion 3D printer to develop a Janus piezoelectric patch. The top layer of the patch is poly(ethylene glycol) diacrylate (PEGDA) loaded with piezoelectric nanocomposite material, which plays a sonodynamic antibacterial effect, and the bottom layer is methacrylate gelatin (GelMA) loaded with vascular endothelial growth factor (VEGF), which can accelerate wound healing (Figure 7A,B) [66]. Chronic diabetic wounds are extremely difficult to heal due to high inflammation, high oxidative stress, and a high-sugar environment [85]. Among them, the high-sugar microenvironment makes the wound susceptible to bacterial infection. Antibiotics remain the most effective treatment, but their abuse can further exacerbate the production of bacterial resistance. Although many antibacterial hydrogels have been reported, an ideal dressing for treating chronic wounds remains a significant challenge. Liu et al. developed a smart antibacterial drug release SBMA/HA-GS/CS (SHGC) Janus wound dressing for diabetic wound healing, which can effectively kill *Escherichia coli* (*E. coli*) and *Staphylococcus aureus* (*S. aurues*). (Figure 7C,D) [86]. In this dressing, the combination of HA-GS (hyaluronic acid is grafted with gentamicin sulfate) and zwitterionic polymers (SBMA) can prevent bacterial and protein adhesion, chitosan (CS) is applied on one side of the hydrogel to improve the adhesion to the skin tissue, and an antibacterial hydrogel wound dressing can be obtained. The dual antibacterial strategy, combining physical anti-fouling and chemical sterilization, works together to reduce the risk of infection.

### 4.5. Self-Adhesive Properties and Antifouling Properties

**Figure 8:**
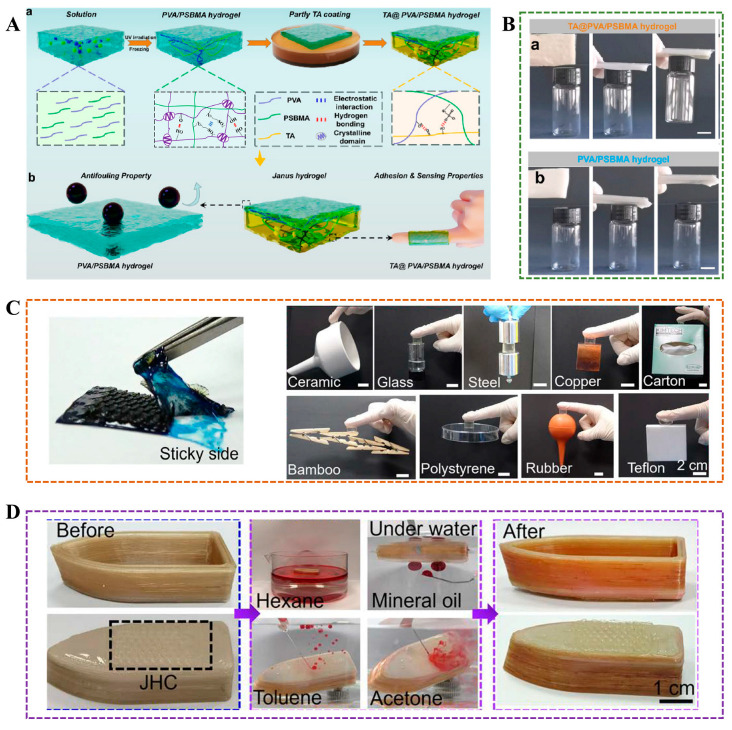
(**A**) Fabrication process and functional applications of PVA/PSBMA Janus hydrogel. (**B**) The asymmetric adhesion properties of PVA/PSBMA Janus hydrogel [72]. (**C**) Adhesion performance of JHC on various substrates. (**D**) Anti-fouling properties of JHC [87].

Janus hydrogel exhibits exceptional self-adhesion and antifouling properties. Its asymmetric structure combines adhesive and non-adhesive faces, enabling strong bonding to various surfaces while resisting the accumulation of unwanted material. This dual functionality ensures reliable performance in diverse environments, making it ideal for applications requiring durable, contamination-resistant interfaces. A representative performance demonstration can be found in ophthalmology, where poly(2-hydroxyethyl methacrylate) (PHEMA) hydrogels are widely used as artificial corneas but face challenges such as poor epithelialization and intraocular deposit formation due to their bio-inertness and relatively smooth surface. In order to solve the problem of poor corneal epithelialization in PHEMA. An effective strategy is to utilize the fact that PHEMA has many exposed hydroxyl groups on the surface, which can be used as the reaction site to modify PHEMA to obtain hydrogels with both anti-fouling and adhesion properties. For example, Han et al. developed a Janus PHEMA film by grafting poly(2-methacryloxyethyl phosphorylcholine) (PMPC) and Arg-Gly-Asp (RGD) peptide groups on both sides of PHEMA, respectively, with anti-fouling properties on one side and cell adhesion properties on the other side, which is expected to be applied in the field of ophthalmology [74]. Beyond biomedical uses, the self-adhesive and antifouling properties also show great promise in industrial and environmental applications. Inspired by the coexistence of fish skin structure and mucus, Zhang et al. designed a Janus hydrogel coating (JHC) composed of poly(acrylic acid-co-acrylamide) and quaternary ammonium chitosan [87]. This coating exhibited a remarkable shear adhesion strength of 103.3 ± 17.5 kPa and a normal adhesion force of 30.97 ± 6.3 mN—ten times greater than that of the non-adhesive side. The adhesive face firmly attaches to various substrates, including ceramics, glass, and steel (Figure 8C), while the antifouling surface demonstrates an extremely low friction coefficient, allowing objects to slide off at a tilt angle as small as 5°. Moreover, the antifouling side resists adhesion to n-hexane, toluene, mineral oil, and acetone (Figure 8D). Although most reported Janus hydrogels exhibit anti-fouling properties, due to the high surface free energy of the hydrophilic layer, redundant adsorption sites remain, resulting in mineral ion adsorption and scaling. Liu et al. developed a novel Janus membrane using a hydrophobic polytetrafluoroethylene substrate and a hydrophilic gel coating prepared by chitfroman ions and alginate, which can address both mineral scaling and organic pollution for seawater desalination [88]. In sensor applications, this anti-fouling property is equally important for maintaining stable signals and ensuring continuous adhesion. Xu et al. designed a TA@PBSMA/PVA Janus hydrogel with excellent anti-fouling performance. The bottom layer of the hydrogel contains TA molecules that can be attached to various substrates and applied in complex environments, such as oil and water environments, for sensing (Figure 8A,B) [72]. Zwitterionic hydrogels play a crucial role in preventing biological contamination and oil pollution applications; however, their weak adsorption to substrates and complex production processes hinder their practical application [89,90]. To overcome these challenges, Zhang et al. developed a self-generating zwitterionic Janus hydrogel coating. Upon contact with water, the top layer hydrolyzes to generate zwitterionic groups, forming a hydrophilic and porous hydrogel that prevents protein adsorption, bacterial attachment, diatom accumulation, and oil fouling. Meanwhile, the lower layer forms covalent bonds and hydrophobic interactions with the substrate, providing a strong adhesion force of up to 7.52 MPa, which is significantly higher than that of traditional hydrogel coatings [91]. This coating has the ability to trigger self-generation by water, and can be applied on various substrates on a large scale through various coating methods such as casting, immersion, brushing, and spraying to protect the substrate from bacterial and oil contamination.

### 4.6. Conductive Properties

Biomaterials with conductive properties can be widely used in various smart devices, such as human health monitoring. Zhang et al. developed a stretchable and highly sensitive porous conductive Janus hydrogel, which is expected to be used in intelligent monitoring devices as a high-performance strain sensor due to its controllable Janus porous structure [92]. Most conductive hydrogels exhibit no difference in electrical conductivity between their surfaces and are conductive as a whole [93]. Such hydrogels are used in flexible sensors and electronic skin fields, and there is the possibility of short circuits or current damage to the skin [93,94]. Ma et al. prepared bilayer hydrogels with or without polyaniline based on PVA, which exhibit asymmetric conductive properties and protect the skin from electrical current damage; however, the electrical conductivity of the conductive layer was not ideal [95]. Zhan et al. used sodium alginate, poly(vinyl) alcohol and tannic acid to prepare a composite hydrogel with asymmetric conductivity, in which the strong conductive layer has a maximum conductivity of 2.95 S·m−1, and the weak conductive layer (less than 0.6 S·m−1) is used as the isolation layer to avoid short circuit and current leakage during use [96].

### 4.7. Biocompatibility

Janus hydrogel exhibits excellent biocompatibility, rendering it suitable for various medical applications. Its asymmetric structure minimizes adverse reactions, while its hydrophilic and hydrophobic components support cell growth and tissue integration. This compatibility ensures safe interaction with biological systems, enhancing its potential for use in the biomedical field. Chen et al. prepared a hydrogel–Janus nanofiber scaffold (NFS) that demonstrated excellent biocompatibility in vitro and in vivo [44]. In vitro cytotoxicity tests using L929 fibroblasts revealed significant cell proliferation, with a survival rate exceeding 150% after 7 days of co-culture, surpassing the control group (116%). Fluorescence microscopy confirmed normal spindle morphology and tight adhesion of fibroblasts to the scaffold surface, indicating no adverse effects on cell viability. Live/dead staining of primary epidermal cells further validated robust cell survival and minimal apoptosis, with no nuclear condensation observed. In vivo studies on full-thickness rat wounds demonstrated accelerated healing, reduced inflammation, and enhanced tissue regeneration. Additionally, the dual antibacterial properties of CMCS-Ag and PCE synergistically mitigated infection risks without compromising biocompatibility. The hydrophobic intermediate layer is composed of polycaprolactone, a biodegradable polymer that supports cell attachment and proliferation. In another study, integrating biocompatible components- gelatin, PLA, and zwitterionic polymers- ensures compatibility with biological systems, supporting its potential for clinical use in hemostatic and anti-adhesion applications [40]. Cytocompatibility assays confirm that J-TP extracts do not impair cell viability, with live/dead staining revealing high survival rates. Hemocompatibility tests show a low hemolysis ratio (1.41%), well below the ASTM safety threshold of 5%.

### 4.8. Others

In the clinical field of orthopaedics, most implants are made of metal; however, the internal liquid environment can corrode the implants, leading to further infection and rejection, and posing a threat to human health. Jian et al. employed one-step electrodeposition to develop a chitosan coating with a smooth and rough asymmetrical structure, which can reduce the corrosion rate of commonly used orthopaedic grafts, such as titanium and stainless steel, and has excellent application prospects in the field of implant corrosion resistance due to its simple preparation [97]. Good breathability improves user acceptance and comfort for some medical products, such as wearable devices. A thin and consistent Janus membrane, consisting of a hydrophilic nanofiber membrane and a hydrophobic porous membrane, is used for pH sensors, ensuring comfortable and reliable wearable applications due to its sweat-wicking ability and breathability [98].

## 5. Janus Hydrogel Applications

**Scheme 2:**
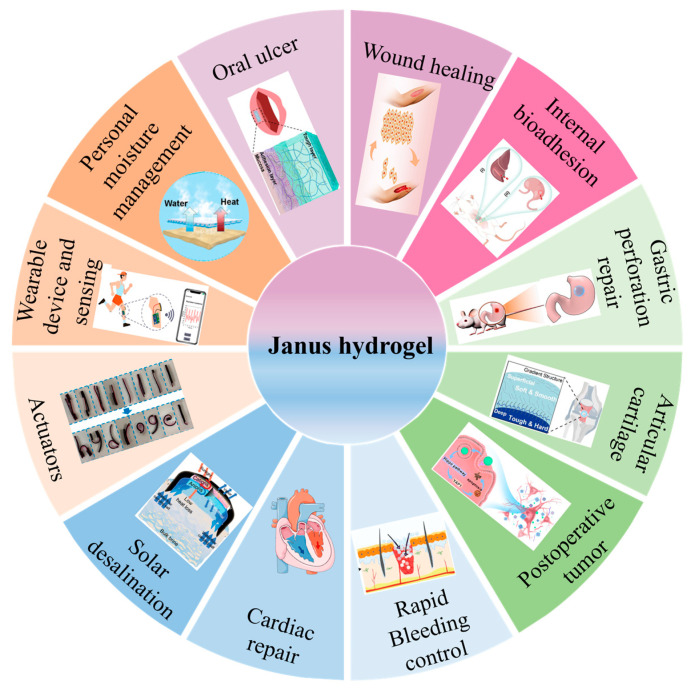
Diagram of Janus hydrogel’s applications.

Janus hydrogels are advanced materials characterized by their asymmetric architecture and multifunctional capabilities, demonstrating significant applicability across various fields in recent years. These hydrogels are utilized in drug delivery mechanisms, tissue engineering, and wound management in the biomedical arena due to their superior biocompatibility and regulated drug release profiles. The unique asymmetric design facilitates targeted drug administration, thereby enhancing therapeutic outcomes while mitigating adverse effects. In environmental science, Janus hydrogels play critical roles in water purification and ecological restoration, including the adsorption of heavy metal ions and organic contaminants. Their distinctive surface characteristics render them exceptionally effective for pollutant separation and degradation. In seawater desalination efforts, the asymmetric wettability of Janus hydrogels facilitates efficient water evaporation and salt separation, offering innovative solutions to freshwater scarcity challenges. Furthermore, these hydrogels exhibit significant utility in braking systems and wearable technologies. Their rapid responsiveness to external stimuli, such as temperature fluctuations, pH variations, and light exposure, makes them ideal candidates for soft robotics and micro-mechanical apparatus braking materials. In smart wearables, Janus hydrogels can be harnessed to create flexible sensors capable of real-time monitoring of physiological parameters (for example, heart rate, body temperature), thus providing crucial technological support for health management. The versatility and intelligent design of Janus hydrogels indicate extensive prospects for application across diverse fields. This section will elucidate the multifaceted applications of Janus hydrogels in various disciplines (Scheme 2).

### 5.1. Wound Healing

**Figure 9:**
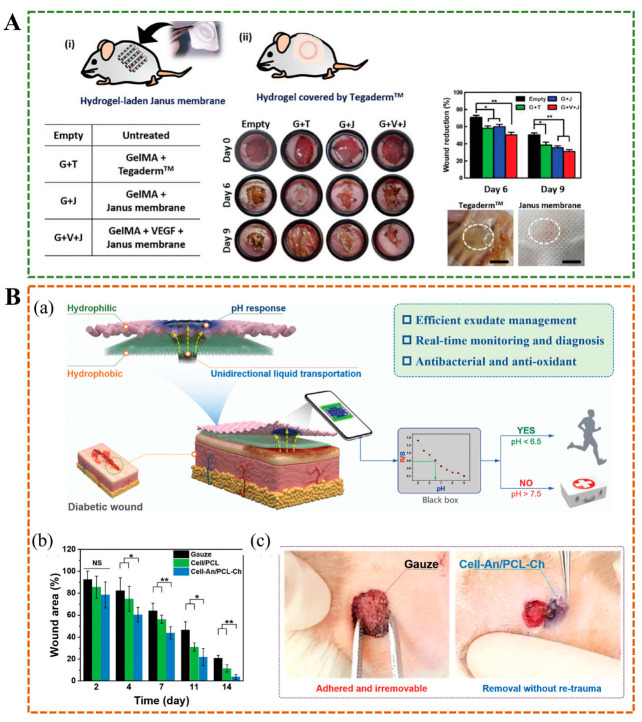
(**A**) Functionalized Janus hydrogel promotes wound healing [99]. (**B**) (**a**) Schematic illustration of Janus hydrogel applied to diabetic wound healing. (**b**) Wound healing degree after treatment with different dressings. (**c**) Removal of gauze and cell-An/PCL-ch hydrogel at the wound site. * *p* < 0.05, ** *p* < 0.01, NS (not significant) [25].

The dressing applied to the skin wound should not only protect against infection from the external environment but also retain moisture at the wound site to optimize therapeutic outcomes. Janus hydrogel shows dual functionalities, presenting significant potential in dermatological applications. An et al. used initiated chemical vapor deposition (iCVD) technology to coat a polyester film with a fluoropolymer film, featuring a hydrophilic carboxyl group on one side. The methacrylate gelatin, coated with growth factor, was fixed to the hydrophilic surface. The resulting Janus hydrogel exhibited good antibacterial, waterproof, and breathable properties, comparable to those of commercially available film dressings like Tegaderm, in promoting wound healing (Figure 9A) [99]. Maintaining a balanced moisture level in the wound area is critical for promoting wound healing and tissue regeneration. Chen et al. designed a hydrogel Janus nanofiber scaffold self-pumping dressing. In this scaffold, the CMCS-Ag gel layer acts as the inner layer to absorb wound exudate, and the middle layer PCL and outer layer PCL-PCE promote the exudate drainage, which allows for unidirectional fluid transport, sustaining water balance at the dressing interface and promoting comprehensive wound repair and skin regeneration [44]. The synthesis of numerous Janus hydrogels involves complex procedures, and challenges related to interlayer bonding can compromise the controllability and reproducibility of the hydrogels, hindering their scalability for practical application. Guo et al. introduced a straightforward method for fabricating Janus hydrogels by controlling the migration of functional groups in waterborne polyurethane through the evaporation of emulsion moisture. This approach resulted in hydrophilic components, such as quaternary ammonium salts, being concentrated on one side, while hydrophobic components remained on the opposite side, yielding an asymmetric hydrogel with a fivefold disparity in adhesion strength between the surfaces. The hydrogel exhibited excellent hemostatic properties and effectively closed wounds in animal models; the incorporation of quaternary ammonium salts also facilitated the healing of infected wounds [100]. The wound healing process is intricate, particularly as the pH of tissue exudate varies throughout different stages of diabetic wound healing [101]. Developing an intelligent dressing that provides unidirectional drainage and monitors the healing process is of considerable practical importance. Xu et al. designed cellulose-anthocyanin/polycaprolactone-chlorhexidine (cell-An/PCL-ch) Janus hydrogels, which exhibit asymmetric hydrophilicity, allowing for unidirectional drainage of sugar exudates and employing pH monitoring to track the wound healing process, indicating promising applications in managing diabetic wounds (Figure 9B) [25].

### 5.2. Internal Bioadhesion

**Figure 10:**
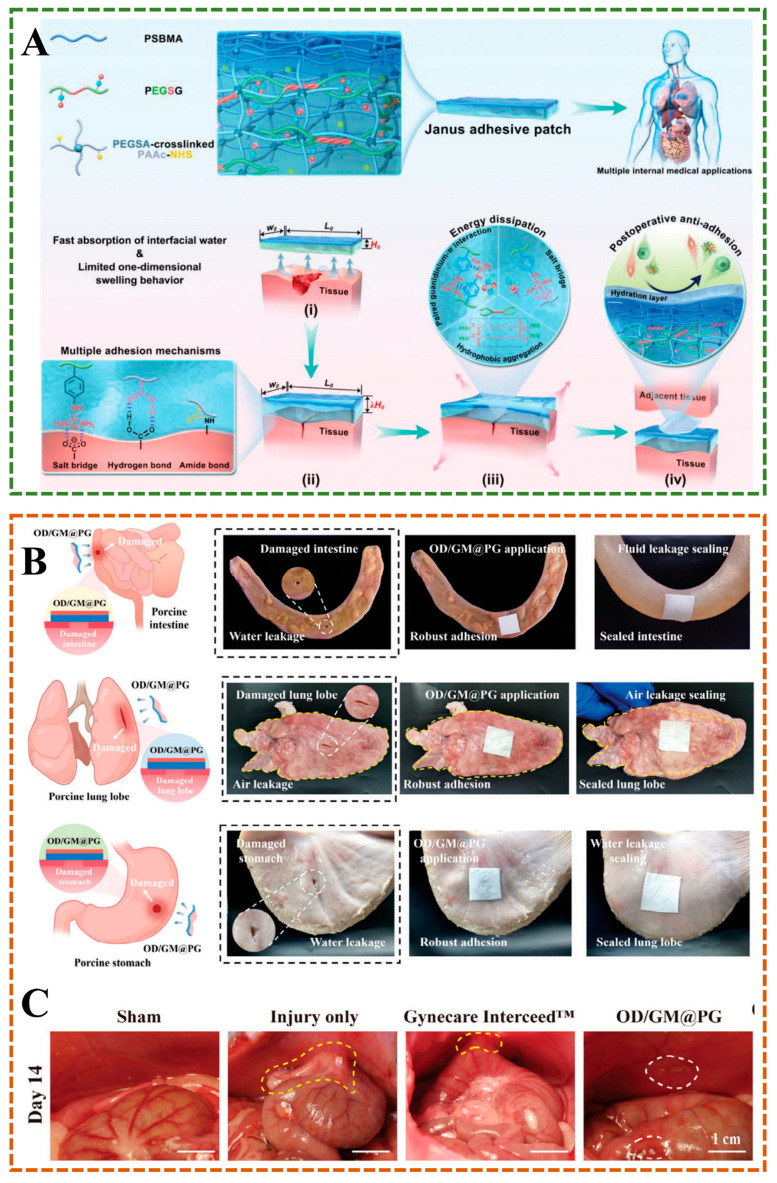
(**A**) Janus hydrogels are designed for application to internal adhesion [21]. (**B**) The Janus hydrogel seals pig intestines, lungs and stomach while preventing postoperative adhesion. (**C**) Postoperative anti-adhesion performance of OD/GM@PG hydrogel [103].

Traditional tissue adhesives, including surgical sutures and staples, often struggle to perform effectively in dynamic and humid internal environments, such as blood and interstitial fluid, or in the cyclical nature of cardiovascular activity [102]. Hydrogels with weak adhesion to wet tissue easily detach from the wound. However, modifying polymers to increase adhesion between hydrogels and tissues through methods such as covalent binding, electrostatic interactions, hydrogen bonding, catechol binding, and topological entanglement may lead to post-operative adhesion and secondary injury. A study has developed a biological patch with ultra-high, long-lasting, and reliable adhesion strength, as well as post-operative anti-adhesion properties. The Janus adhesive patch is prepared by physically cross-linked guanidinylated PEGylated ply(glycerol sebacate), and acrylylated PEGylated ply(glycerol sebacate) as a macromolecular crosslinking agent, chemically cross-linked with poly(acrylic acid)-N-hydrosuccinmide ester, and further interpenetrated with single-sided zwitterionic polymer layer, which had excellent water absorption ability and limited one-dimensional expansion behavior (Figure 10A) [21]. Lv et al. developed a double-layer biomimetic microstructure OD/GM@PG Janus hydrogel. In this hydrogel, the inner layer is theophenol-modified oxidized hyaluronic acid (OD) and methylacrylyl gelatin (GM), and the outer layer is gelatin and polycaprolactone (PG), the two layers are tightly bonded through covalent/non-covalent crosslinking, which can robustly seal water-leaking intestine/stomach and air-leaking lung, while preventing abdominal and intrauterine adhesion and promoting endometrial healing in animal models(Figure 10B,C) [103]. Wang et al. made a hydrogel with Janus structure by controlling the distribution of free carboxyl groups, which were mainly distributed on the bottom surface of the hydrogel, resulting in a 20-fold difference in adhesion strength between the top and bottom surfaces of the hydrogel [104]. The rabbit model experiment showed that the bottom surface of the hydrogel was closely bonded to the stomach tissue, while the poor adhesion of the top surface could prevent postoperative adhesion, effectively promote gastric injury repair. In situ Janus hydrogels are attractive for their ease of delivery and ability to provide complete coverage of irregular tissue surfaces. Jia et al. designed a sprayable Janus powder to form a gel barrier in situ, allowing it to adapt to complex and dynamic organ movements and prevent postoperative adhesion [105]. The Food and Drug Administration approves the powder ingredients for clinical use, and they are easy to mass-produce and store, which has great potential in clinical transformations, such as endoscopic surgery [105].

### 5.3. Gastric Perforation Repair

The most common cause of gastric perforation is peptic ulcers, which can also be caused by ruptured stomach cancer and surgical procedures, and can lead to toxic shock in patients, which is life-threatening [106]. Wu et al. grafted catechol onto hyaluronic acid, and the resulting injectable Janus HAD hydrogel exhibited asymmetric properties of adhesion and anti-adhesion, which could be combined with a minimally invasive, deliverable device to repair gastric perforation in a pig model and prevent postoperative adhesions [107]. In actual clinical practice, the situation of gastric perforation is more complicated, often presenting as a tilting and bending state. The hydrogel, with poor adhesion, makes it difficult to stay in place on the wound and cannot achieve an effective sealing effect. On the basis of HA grafting DA, the same research group introduced phenylboric acid (PBA) to develop a new Janus hydrogel for treating gastric perforation [22]. Due to the formation of a borate ester bond, the hydrogel exhibited self-healing properties and shear thinning behavior, which addressed the issue of limited residence time in complex wounds and maintained good injection performance. After subsequent photocuring, the hydrogel still prevents postoperative adhesion [22]. Compared to linear polymers, a high grafting amount of catechol polymers can enhance adhesion strength and reduce bonding time. Liang et al. developed an asymmetric adhesive Janus sealant composed of PAA crosslinked by PEG−600 and gelatin crosslinked by catechol-modified hyper-branched polymer. This sealant overcame swelling or hydrolysis behavior in an acidic environment and interface fatigue caused by a dynamic environment, and had immediate wet adhesion performance, effectively prevented postoperative adhesion, which is expected to be applied in gastric perforation repair and other related medical fields [108].

### 5.4. Wearable Devices and Sensors

Traditional hydrogel sensors are inconvenient to use due to the need for additional tape fixation. The strong adhesion properties of polyampholyte hydrogels can solve this problem [109,110]. However, due to the non-different adhesion properties of both sides, the exposed side can adhere to other impurities, which affects data acquisition. Wang et al. prepared a flexible strain sensor with single-sided adhesion performance by cyclic freeze–thaw treatment of a poly(vinyl alcohol)/polyaniline solution and poly(vinyl alcohol)/phytic acid, which can be integrated with a Bluetooth system to monitor various physiological activities wirelessly [73]. Sensors widely utilize anisotropic particles due to their high sensitivity, selectivity, and unique coding ability [76]. Some natural polymers, such as alginate and cellulose, are frequently utilized in sensor development due to their low cost and excellent biocompatibility. Hou et al. used sodium alginate and sodium alginate loaded with different concentrations of silica to develop a Janus microfiber with reversible coiled/uncoiled performance under a humidity gradient, utilizing microfluidic spinning technology to achieve flexible and controllable red, yellow, and green LED lights for the preparation of humidity-sensing intelligent switching devices [111]. Additionally, traditional hydrogels with poor toughness and mechanical properties fail to expand further in the application of flexible sensors. Sun et al. grafted polypyrrole (PPy) and polydopamine (PDA) asymmetrically on different sides of cellulose nanocrystals (CNCs) and then combined the obtained Janus CNCs-PPy/PDA (JCNs) with polyacrylic acid to prepare hydrogel with good self-healing properties, which also had the advantages of stable detection signal and high sensitivity, and can monitor a variety of human movements such as swallowing, frowning and joint movement [112].

### 5.5. Postoperative Tumor Prevention

Surgical resection is the most commonly used method for the treatment of bone tumors and malignant melanoma, but preventing postoperative recurrence and promoting postoperative tissue repair has always been an urgent clinical problem. Huang et al. designed a core–shell hydrogel bone scaffold that can release melatonin programmed. The shell was GelMA loaded with high melatonin, which is responsible for releasing high melatonin quickly in the early stage and removing residual tumor cells; the core was HAMA and F127A loaded with low melatonin concentration, which was responsible for releasing low melatonin concentration in the later stage and promoting bone repair [113]. The Mel@Gel/Mel@HF hydrogel with a Janus structure provides a safe and effective solution for the postoperative management of bone tumors [113]. A hypoxic microenvironment will further aggravate tumor recurrence and metastasis, but also damage angiogenesis and seriously affect wound healing. Chen et al. designed a sprayable hydrogel loaded with nanomedicine and cyanobacteria [114]. Cyanobacteria can supplement oxygen through photosynthesis, promote oxidative stress in nanomedicine-induced tumor cells, further prevent tumor recurrence and metastasis, and promote wound healing [114]. This oxygen-induced Janus-regulated hydrogel has broad application prospects in postoperative tumor treatment.

### 5.6. Rapid Bleeding Control

**Figure 11:**
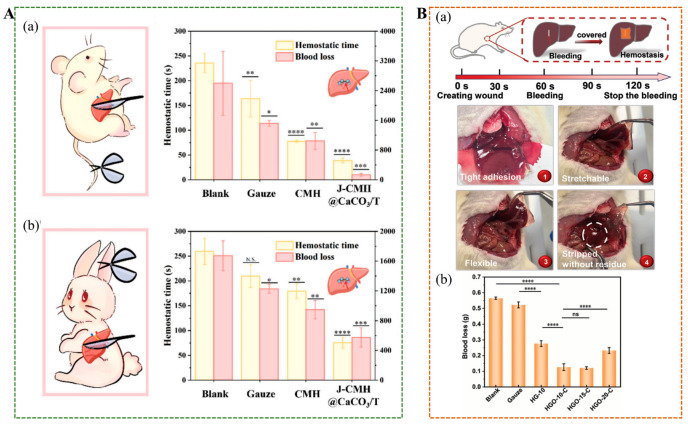
(**A**) (**a**) Hemostatic time of hepatic hemorrhage was treated with different samples in rat hepatic hemorrhage model. (**b**) Hemostatic time of hepatic hemorrhage was treated with different samples in rabbit hepatic hemorrhage model [115]. (**B**) (**a**) Hemostatic results of hydrogel applied to the rat liver injury model. (**b**) Quantitative blood loss of rats treated with gauze and HGO-C Janus hydrogels. * *p* < 0.05, ** *p* < 0.01, *** *p* < 0.001, **** *p* < 0.0001, ns for no significant differences [116].

Uncontrolled bleeding caused by accidents, surgeries, and diseases is one of the major causes of death. Traditional materials, such as tourniquets and gauze, are often difficult to adapt to incompressible and irregular wounds, frequently failing to achieve the desired results. Yu et al. crosslinked part of calcium ions with carboxylated chitosan (CCS), made CaCO_3_ distribute unevenly in the sphere due to gravity sedimentation [115]. Then irradiated with ultraviolet light, and removed the CCS shell to obtain Janus J-CMH@CaCO_3_/T hydrogel with excellent self-propelled performance, which has a strong ability to store and release calcium ions, and could effectively improve hemostasis efficiency, and stop bleeding within 39 s in rat tail and liver haemorrhage models, hemostasis can be achieved within 109 s in the rabbit model of the auricular artery and liver haemorrhage (Figure 11A). Peng et al. developed a Janus tissue patch (J-TP) with rapid hemostasis and prevention of postoperative tissue adhesion, in vivo evaluations confirmed J-TP’s efficacy in sealing bleeding wounds with a high burst pressure (312.5 mmHg) and minimal postoperative tissue adhesion [40]. The patch reduced blood loss in rats in a model of liver bleeding by 66% compared to commercial glue. Sun et al. synthesized HGO-C Janus hydrogel with asymmetric adhesion properties using natural polymers, containing an adhesion layer (HGO) and an antiadhesive layer (CGC) [116]. Unlike the easily oxidizable catechol structure, tris(hydroxymethyl)aminomethane improved the hydrogel adhesion, while negatively charged natural polysaccharides, such as dextran and hyaluronic acid, contributed to its strong resistance to burst pressure. These combined properties effectively accelerated the hemostatic process (Figure 11B) [116].

### 5.7. Cardiac Tissue Repair

Myocardial infarction is a significant threat to human life and health, and many medical materials used for heart repair may cause secondary damage to the myocardium. Compared to abdominal wall and gastric perforation repair, cardiac tissue is more complex and challenging. Myocardial infarction is accompanied by the production of a large number of reactive oxygen species, which leads to further inflammation, necrosis, and fibrosis, hindering cardiac repair and regeneration. Some Janus binders have asymmetric adhesion properties, which can prevent postoperative tissue adhesion problems. However, due to the lack of antioxidant, anti-inflammatory, mechanical, and electrical conductivity properties, it is difficult to achieve the function of cardiac repair. He et al. developed a Janus CPAMC/PCA cardiac patch with excellent electrical conductivity, antioxidant, anti-inflammatory, biocompatible, and mechanical properties [39]. Featuring dual properties of fast adhesion and anti-adhesion, the patch can be removed on demand, preventing tissue adhesion after myocardial infarction and postoperative secondary trauma, making it highly suitable for myocardial infarction repair.

### 5.8. Articular Cartilage Regeneration

On one side of the articular cartilage is a soft layer that is highly hydrated to protect against sliding friction, and on the other side is a calcified area that tightly connects the cartilage to the bone. The lack of a Janus structural and functional substitute makes it challenging to apply to cartilage transplantation. Luo et al. designed a Janus hydrogel using sodium hyaluronate, chitosan, PVA, and hybrid hydroxyapatite, which exhibited a friction coefficient as low as 0.024 and showed potential for bone integration, making it a promising candidate for use as a cartilage substitute in tissue engineering [117].

### 5.9. Soft Actuators

Actuators are utilized in a wide range of applications, including microrobotics, healthcare, and drug delivery. However, developing intelligent actuators with fast response capabilities is still challenging. Li et al. developed a double-layer Janus actuator composed of a thermal dual-network hydrogel and elastomer, which exhibited high interface toughness and controllable shape deformation, providing a new idea for designing and applying soft electronic devices [118]. Although the bilayer Janus structure can be used to fabricate actuators, interfacial delamination restricts further deformation and cycling of the actuators [119]. Wang et al. employed the “self-generation” method to construct interpenetrating network (IPN) interfaces, providing a solution to the problem of interlayer peeling in Janus hydrogels, thereby enhancing interface adhesion and cycle durability [120]. Through the combination of the temperature-sensitive poly(*N*-isopropylacrylamide) (PNIPAM) layer and the photothermal polyacrylamide-Graphene oxide (PAM-GO) layer, a rapid and reversible response to temperature and near-infrared light was achieved, and precise bending deformation up to 360° could be accomplished. Preparation of single-layer Janus hydrogel actuator is also a method to address the instability of interlayer bonding. Ren et al. developed a simple, degradable and sustainable single-layer Janus LCNF/PVA-AT membrane by adjusting the distribution of lignin-containing cellulose nanofibrils (LCNP) in PVA and further alkaline treatment (AT) [121]. The response/recovery time and deformation degree can be controlled by adjusting the salt type, concentration and other factors, with high sensitivity and intelligent controllability, and has a broad application prospect in the preparation of multifunctional actuators.

### 5.10. Biomarker Detection

Janus hydrogels exhibit excellent performance in biomarker detection due to their asymmetric double-sided structure. One side is highly enriched with target molecules, and the other side realizes signal conversion, significantly improving detection sensitivity and selectivity. Its multifunctional interface is compatible with various detection modes (such as electrochemical and optical) and is suitable for high-precision analysis of proteins, nucleic acids, and other markers, providing an efficient and portable new platform for early disease diagnosis and real-time monitoring. MicroRNAs, as potential biomarkers for early diagnosis, can be used to monitor a variety of diseases. However, due to the low concentrations and short lengths of microRNAs, the commonly used detection techniques have several problems, including complexity, time consumption, and high cost. Lim Jaewoo et al. developed a Janus hydrogel using a signal amplification strategy for fuel-assisted DNA cascades, achieving high sensitivity that promises to be a new technology for early cancer detection and prognostic monitoring [122]. The hydrogel integrates fuel stimulant-powered (FSP) amplification, enabling enzyme-free, temperature-independent fluorescent signal enhancement via a DNA cascade reaction. This system immobilizes distinct FSP probes, each containing FAM/BHQ1 for miR-135b and Cy3/BHQ2 for miR-21, on separate hydrogel regions, allowing simultaneous, independent detection of multiple targets. With a detection limit of less than 10 fmol, the platform exhibits high sensitivity and specificity, as validated using synthetic miRNAs, cell lines, xenograft mouse models, and clinical human sera. In clinical samples, Janus hydrogels effectively distinguished gastric cancer patients (stages I–IV) from healthy donors by quantifying miRNA overexpression. The hydrogel’s 3D architecture minimizes background noise and enhances probe accessibility, while its scalable fabrication and low-cost design support point-of-care diagnostics.

### 5.11. Personal Moisture Management

**Figure 12:**
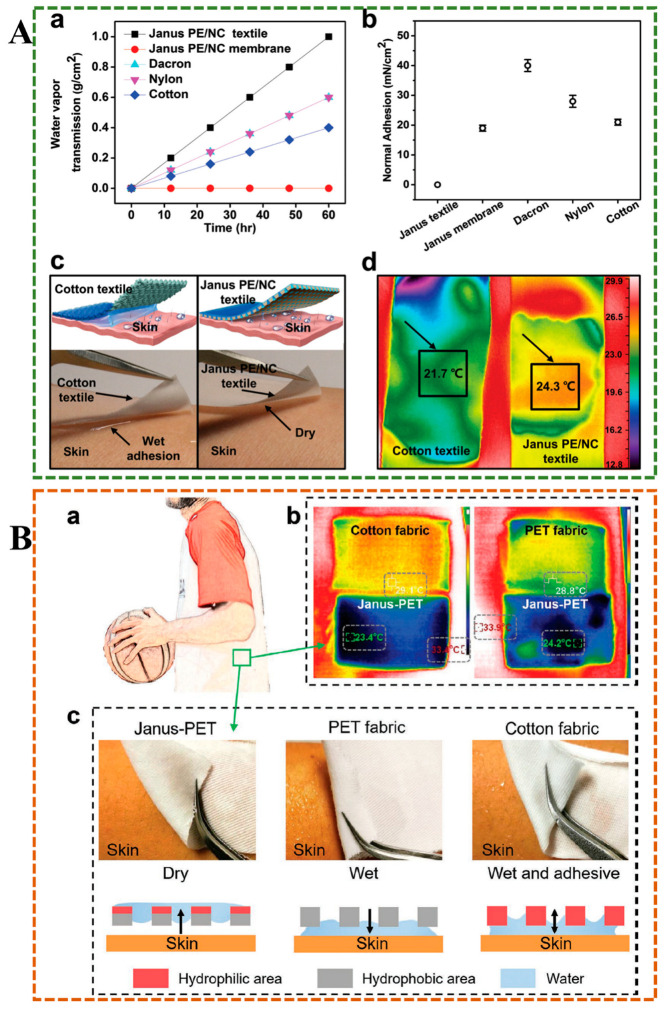
(**A**) The wearability test of Janus PE/NC textile. (**a**) Tests on the water vapor transmission rates of various materials. (**b**) The adhesion of different wetted fabrics to the skin. (**c**) Images of the Janus PE/NC textile illustrate its rapid liquid transport capability. (**d**) Thermal imaging compared the temperature profiles of the Janus PE/NC textile and a conventional cotton textile applied to moist skin [123]. (**B**) Application test of hydrogel in sweat absorption management of sports clothing. (**a**) The application illustration of the Janus PET fabric. (**b**) Thermal images compared the PET, cotton, and Janus PET fibric. (**c**) The rapid drying and moisture-wicking properties of Janus PET fabric [124].

Moisture management is one of the most vital criteria for evaluating functional textiles. Excessive sweat produced by the skin can cause uncomfortable sensations, such as feeling sticky and cold. Cotton fabrics have the ability to absorb sweat but cannot achieve efficient delivery of sweat, resulting in water accumulation. Dai et al. have developed a textile with both hydrophobic (polyester, PE) and superhydrophobic (nitrocellulose, NC) asymmetrically porous structures through laser drilling, maintaining the water absorption and air permeability of traditional fabrics, with a directional water transport capacity of up to 1246%, preventing the stickiness and cold caused by excessive sweat (Figure 12A) [123]. Miao et al. designed a three-layer PU/(PU-HPAN)/HPAN fiber film, which had progressive wettability characteristics, enabling the fiber film to carry out water transport spontaneously, continuously, and in a directional manner, and prevent penetration in the reverse direction, can be used in the textile field to bring dry and comfortable experience to the wearer [42]. However, these studies focused on the directed delivery of water, ignoring the bacterial growth caused by sweat, which can further lead to skin and other diseases. Wang et al. used a simple dip-pad-cure method to design a Janus poly(ethylene terephthalate) (PET) fabric treated with cation-π hydrophilic agent (CPHA) on one side, which had a unidirectional water transport capacity of 1115%, maintained skin dry and comfortable, and also had good antibacterial properties (Figure 12B) [124]. As a novel functional textile, it not only solves the problem of moisture management but also considers human health and hygiene, which has excellent potential for application. Janus fabrics are easily damaged during use and cannot withstand multiple home washes, which affects their one-way water transport capacity. Zhou et al. employed a simple and rapid thermal transfer/laser method to produce Janus fabric with exceptional moisture management capabilities, which can withstand over 900 friction cycles and 250 household laundry cycles, exhibiting good durability [125].

### 5.12. Smart Textile Fabrication

With the change in extreme environments, people expect to adjust the temperature of the human body through clothing, and smart textiles with thermal regulation performance are a future development trend. Most of the radiation-cooled textile materials reported in the study cannot be intelligently adjusted to the appropriate temperature in response to temperature changes [126]. Xie et al. prepared an intelligent Janus silver nanowire/reduced graphene oxide/poly(vinylidene fluoride-co-hexafluoropropylene) (AgNWs/rGO/PVDF-HFP) film with controllable thermal regulation using the asymmetric chemical assembly method of fiber spinning [127]. The two sides of the film showed opposite photothermal properties, which can adjust the textile to perform intelligent selection and switch of thermal insulation and cooling modes according to changes in the external environment.

### 5.13. Cosmetics Production

The commonly used masks are non-woven fabrics and other materials soaked in essence. As an effective cosmetic applied to the face, the development of a spray-on paper removal mask to replace traditional masks has significant environmental benefits. Cai et al. encapsulated the bioactive substance nicotinamide with a whitening effect by the amphiphilic copolymer composed of the hydrophilic material (poly (ethylene glycol), PEG) and the hydrophobic material (poly (ethylene glycol), PLGA), and synthesized the Janus film that can be formed in situ [128]. After being sprayed onto the skin, due to the temperature change, the air side forms a physical hydrogel to keep the facial mask moist, while the skin side forms a suspension gel to facilitate the release of nicotinamide.

### 5.14. Solar Desalination (Solar Water Evaporation)

Water scarcity poses a serious threat to human life. As a low-cost, low-energy, environmentally friendly, and sustainable freshwater production technology, solar-driven interface evaporation is a promising solution to the problem of water scarcity [129]. Chen et al. first proposed the preparation of Janus PGRO@GO (partially reduced graphene oxide@graphene oxide) evaporator by solar irradiation, which can exhibit extremely strong desalination and purification performance in seawater, sewage, and extreme environments such as acid/alkaline water, and can achieve nearly 100% (99.98%) energy efficiency in a single solar irradiation [130]. Another study used a hydrophilic polyacrylamide, a thermal response material poly(*N*, *N*-diethylacrylamide) and a photothermal material CuS to develop a thermoresponsive hydrogel evaporator (CSAm) that can spontaneously form Janus structure under sunlight [131]. Under daytime light, the top of the hydrogel forms a hydrophobic structure, while the bottom is a hydrophilic structure, which reduces heat loss and promotes evaporation performance, at night, the hydrophilic evaporator surface promotes brine transport and enhances salt tolerance. In addition to carbon-based materials and CuS as photothermal materials, Fe3O4 particles possess both photothermal conversion capabilities and magnetic properties, enabling efficient water evaporation through photomagnetic characteristics. Wang et al. used a magnetic positioning strategy to synthesize Fe3O4 and carbon-based materials into Janus structure in poly(vinyl) alcohol (PVA) and polystyrene sulfonate (PSS) hydrogels to prepare an efficient self-cleaning solar evaporator, which could reach the evaporation rate of 3.43 kg m^−2^h^−1^ under single sunlight irradiation and had broad application prospects in long-term solar desalination and wastewater treatment [132]. In addition to the photohot water gel, the amphiphilic Janus patch graft hydrogel developed by Zhu Jie et al. had a thin hydrophobic top layer and a higher ionic strength of the quaternized poly(4-vinylpyridine) entangled layer so that it exhibited excellent water evaporation rate and salt ion rejection ratio, comparable to photothermal hydrogels and superior to all non-photothermal hydrogels [133].

### 5.15. Solar Thermal Desorption

Appropriate air humidity is very important for human health and machine maintenance. We typically use ventilation, heating, and condensation to achieve dehumidification, but these methods have the disadvantages of low efficiency and high energy consumption. Some hygroscopic materials, such as activated carbon and silica gel, have poor dehumidification stability and reusability. Chen et al. developed an environmentally friendly Janus-type hygroscopic hydrogel by freeze-drying and spraying lithium chloride, cellulose nanofibrils, and graphene oxide-based photothermal composites (GMC) [134]. Due to the excellent thermal desorbing properties of graphene oxide-based photothermal composites, the hydrogel can be reused. A Janus-type cellulose nanofibrillated hydrogel (CGH) integrated with a GMC exhibits exceptional solar-driven dehumidification. The asymmetric design combined a porous 3D cellulose network (loaded with hygroscopic LiCl) and a GMC photothermal layer, enabling efficient moisture capture (459.3% weight absorption) and solar-triggered desorption (82.5% water release under 8 h of light). GMC’s full-spectrum solar absorption (200–2500 nm) derived rapid interfacial heating (50 °C within 100 s), synergizing with the LiCl dissolution exotherm to accelerate dehydration. Optimal GMC loading (CGHs5: 5 mg·cm^−2^) balanced photothermal conversion and capillary water transport, reducing dynamic humidity from 97.1% to 39.2% in airflow. Excessive GMC hindered water escape, while insufficient GMC limited heating. Light intensity enhanced the dehydration rate, highlighting solar adaptability. The Janus structure’s dual functionality—moisture retention and energy-efficient regeneration—positions it as a sustainable solution for reusable dehumidification in both static and dynamic environments.

### 5.16. Supercapacitors

Janus hydrogel exhibits exceptional potential in supercapacitors due to its dual-functional asymmetric architecture. The conductive layer, such as polymers/graphene, enables rapid electron transfer, while the porous hydrophilic phase facilitates efficient ion diffusion. This synergy enhances energy/power density and cyclic stability. Its mechanical flexibility further supports integration into wearable electronics, offering sustainable, high-capacity energy storage solutions. Zhang et al. prepared a Janus electrode with dual conduction/pseudo-capacitance function based on Polypyrrole (PPy)-modified graphene oxide (rGO), and demonstrated the ultra-high specific capacitance of 1380 mF cm^−2^ at 1 mA cm^−2^ [135]. The supercapacitors were created to exhibit a peak energy density of 31.95 Wh L^−1^ and maintain 89.7% of their capacitance after undergoing 10,000 charge–discharge cycles. Compared to traditional energy storage systems, this supercapacitor based on Janus hydrogel offers higher energy/power density, as well as improved durability, and is lightweight, portable, and convenient for the wide application of smart devices. Traditional conductive hydrogels are difficult to adapt to in a low temperature environment below zero. Because the water molecules in hydrogels freeze at low temperatures, the cold environment limits the practical application of supercapacitors. The study by Fan et al. highlighted the potential of Janus polyhedral oligomeric silsesquioxane (POSS)-based hydrogels for low-temperature supercapacitors, offering notable advantages such as exceptional anti-freezing performance (1445% stretchability and 0.067 S cm^−1^ ionic conductivity at −20 °C), achieved through synergistic effects of sulfonate groups and LiCl to suppress ice formation [136]. The all-in-one design minimized interfacial resistance and ensured stability under deformation (>90% capacitance retention). However, the synthesis involved complex, multi-step reactions (for example, thiol click chemistry and covalent crosslinking), which raised concerns about scalability and cost. While the hydrogel exhibited robust cycling stability (80% capacitance retention after 2000 cycles), long-term durability in extreme environments and practical scalability remain unaddressed.

### 5.17. Human–Machine Interfaces

Flexible wearable devices can overcome the incompatibility between traditional rigid devices and tissues, serving as a better bridge for the human–machine interface, monitoring physiological signals, and aiding in disease diagnosis. Wei et al. prepared a Janus hydrogel by using gallic acid-modified fish skin gelatin as the bottom layer and pig skin gelatin/glycerin as the top layer through sol–gel hot “welding” to form a double layer interface, which has asymmetric adhesion properties and is expected to be applied to the human–machine interface to achieve accurate measurement [137].

### 5.18. Others

In the field of tendon repair, Janus hydrogels have demonstrated significant advantages due to their unique asymmetric structure and multi-functional integration capabilities. Ouyang et al. successfully constructed a Janus hydrogel with temperature-adjustable properties through a one-step method [138]. This hydrogel exhibited excellent mechanical compliance in the spatial dimension and unilateral adhesion behavior, providing effective mechanical support and physical barriers to prevent postoperative adhesion of surrounding tissues in damaged Achilles tendons. In the temporal dimension, the ROS-responsive prodrug macromolecule effectively eliminates reactive oxygen species (ROS) and alleviates early inflammatory responses, thereby promoting high-quality tendon healing. High-modulus hydrogels are often accompanied by reduced hydration properties, resulting in poor lubrication properties. The environment where articular cartilage is located is humid, and bionic materials are required to have stable adhesion properties. At the same time, materials with soft yet strong mechanical properties and good lubrication performance can better replace articular cartilage for clinical applications. Wan et al. combined a poly(acrylic acid-co-acrylamide p(AAc-co-AAm) hydrogel with a poly(dopamine methylacrylamide-hydroxy-methoxyethyl acrylate) p(DMA-co-MEA) hydrogel to develop a new type of Janus patch with lubricating properties, low friction, fatigue resistance, and high adhesion strength for underwater application [139]. As a common oral disease, oral ulcers significantly impact the quality of life and cause pain in patients [140]. However, clinical treatments include patches and sprays easily removed from the dynamic, moist oral environment [67]. Although the strong adhesive hydrogel can solve the problem of patch shedding, it introduces a new problem-tissue adhesion. The Janus hydrogel prepared by Hongsheng Liu et al. not only has strong adhesion, but also can fall off by itself after oral administration to prevent adhesion, which is a promising method for the treatment of oral ulcers [19]. The content of water in zinc-ion batteries significantly affects the stability of the battery cycle. Zhu et al. designed a Janus hydrogel electrolyte with different hydrophilic and gradient pore structures, which enabled the battery cathode and anode to meet their respective water content requirements and promoted the cycle life of the zinc-ion battery [23]. The side with high hydrophilicity ensured the cathode had enough hydrogen ions, while the side with poor hydrophilicity weakened the anode corrosion.

## 6. Future Outlook

Although Janus hydrogels have achieved considerable progress, their broad application in biomedical, environmental science, and healthcare remains challenging. Current preparation techniques are generally more complex than conventional hydrogel cross-linking methods, often requiring high-precision instruments, strict operational procedures, and highly controlled environmental conditions. These factors increase production costs and severely limit the scalability of large-scale fabrication. Moreover, most existing methods struggle to achieve precise control over macroscopic structural features, such as interface clarity, layer thickness ratio, and geometric morphology. For instance, strategies based on external fields (for example, magnetic or centrifugal fields) are challenging to scale up and cannot meet the demands of efficient and uniform mass production. Stepwise polymerization processes are prone to interlayer molecular diffusion, leading to blurred functional regions. Additionally, bilayer structures produced by these methods often exhibit weak interfacial bonding, making them susceptible to delamination, which further restricts the practical utility of Janus hydrogels. There is also a general lack of systematic evaluation regarding the long-term stability and durability of the interfaces. The interlayer connections in Janus hydrogels typically rely on physical interactions (such as hydrogen bonding, chain entanglements) or weak chemical bonds. Under prolonged use or cyclic mechanical loading, especially in environments involving swelling/shrinking or non-uniform stress, these interfaces are at risk of peeling or slippage. Furthermore, existing preparation methods remain inadequate for handling complex-shaped substrates and large-scale expansion, and their environmental adaptability (for example, under extreme temperatures or pH conditions) requires further validation. Balancing the asymmetric properties on both sides of a Janus structure presents another significant challenge, as it is often difficult to optimize two opposing properties simultaneously. Future advances may rely on integrated multi-technology strategies, such as combining microfluidics with 3D printing, to achieve precise design and controllable fabrication of Janus structures across micro-to-macro scales. This would enable the customization of material systems with specific shapes, compositional gradients, and functional distributions. In applications with stringent optical requirements, such as contact lenses and artificial corneas, Janus hydrogels must provide dual functionality while maintaining high transparency. This demands even stricter control over microstructure and surface morphology. Although promising for preventing postoperative adhesions, challenges remain in achieving strong wet-tissue adhesion, matching the dynamic mechanical properties of organs, and enabling controllable detachment behavior. To advance clinical translation, priority should be given to the use of degradable natural raw materials with well-controlled degradation rates. The characteristic asymmetric properties of Janus hydrogels (for example, “hydrophilic–hydrophobic” and “adhesive–nonadhesive”) have demonstrated great potential in various fields, including wound dressings, anti-adhesion barriers, gastrointestinal and cardiac repair, hemostatic materials, antifouling coatings, and desalination. Nevertheless, the field is still in its early stages, requiring further expansion of application diversity and exploration of new functional paradigms. Inspired by nature, the development of intelligent Janus systems capable of responding to multiple external stimuli (for example, light, enzymes, electric fields, temperature) and performing coordinated actions such as deformation, drug release, and signal output will provide a key material platform for next-generation adaptive soft robots and precision medical devices. Another promising direction involves constructing Janus hydrogels with multiple asymmetric functions and intelligently tunable properties tailored to specific scenarios. For example, integrating sensing, actuation, drug delivery, and energy conversion into a single device could enable “perception–feedback–action” loops for applications such as smart wound dressings within closed-loop diagnostic and therapeutic systems. The design and application of Janus hydrogels represent a highly interdisciplinary research topic that integrates knowledge and methodologies from biology, medicine, physics, engineering, and computer science. Therefore, exploring synergies between Janus hydrogels and emerging advanced technologies will be a crucial direction for future research.

## 7. Conclusions

Characterized by their unique asymmetric attributes, Janus hydrogels offer innovative solutions to complex challenges across various disciplines. This paper provides a comprehensive review of the synthesis methodologies, anticipated properties, and practical applications of Janus hydrogels. The primary fabrication techniques encompass layer-by-layer construction, one-pot synthesis, electrospinning, and the induction of external stimuli, each providing tailored strategies for achieving specific functionalities suitable for diverse applications. We further explore the enhanced properties of Janus hydrogels, which expand their utility in sectors such as biomedicine, environmental science, and health management. The discussion culminates in an examination of specific applications across various domains, including, but not limited to, wound healing, internal adhesion, gastric perforation repair, wearable technology, sensing, postoperative oncology, rapid hemostasis, cardiac repair, articular cartilage restoration, brake systems, personal humidity regulation, and desalination. This paper aims to synthesize recent research on Janus hydrogels, thereby contributing valuable insights to relevant fields and fostering the progressive advancement of this area of study.

## Figures and Tables

**Table 1:** Preparation, properties, and application of Janus hydrogels.

Preparation	Component	Property	Cross-Linking Interaction	Application	Ref.
Layer by layer	Poly (sodium acrylate)/poly (N-isopropyl acrylamide) (PSA/PNIPAM)	Asymmetric swelling; multi-responsive	Network interpenetration	Actuator	[18]
Two-step process	*N*-[tris(hydroxymethyl)methyl]-acrylamide (THMA)/chitosan; poly(ethylene glycol) diacrylate (PEGDA) and poly(vinyl alcohol) (PVA)	Wet adhesion performance; self-deboning properties	Ultraviolet light crosslinking	Oral mucosal ulcers	[19]
Thermal polymerization	*N’, N’*-methylenebis(2-propenamide), sulfobetaine methacrylate	Adhesion/anti-adhesion; Hexagonal facet	Free radical polymerization	Prevent postoperative adhesion	[20]
Two-step, light-curing method	PEGS	Adhesion/anti-adhesion; Limited 1D swelling behavior	Monomer diffusion;	Internal bioadhesion	[21]
Two-step, cross-linking process, Light curing	Hyaluronic acid, Dopamine, Phenylboronic acid	Adhesion/anti-adhesion; inherent mechanical strength	Photo crosslinking	Gastrointestinal perforations	[22]
Copolymerization	Acrylamide (AM), Zinc acrylate (Zn-AC), Nisopropylacrylamide (NIPAM) and N, N’- methylenebisacrylamide (MBAA)	Higher hydrophilicity/lower hydrophilicity; gradient pores	copolymerization	Zinc-ion batteries	[23]
Ultraviolet irradiation	patterned polydimethylsiloxanes (PDMS), P(NiPAAmbis- AA) hydrogel film	Hydrophilicity/hydrophobicity	Ultraviolet light crosslinking	Pressure sensors, uterus cervical canal tension perception	[24]
Two-layer	Cellulose-anthocyanin; polycaprolactone-chlorhexidine	Hydrophilicity/hydrophobicity; pH-response;	Electrospinning	Diabetic wound healing	[25]
Three-layer	Polyacrylamide; brominated isobutylene isoprene rubber; polydimethylsiloxane	Moisturize; antimicrobial	Van der Waals force; hydrophobic interaction; hydrogen bond	Preservation of bone relics	[26]
Stacked under vacuum using cyclic freezing	DL-a-tocopherol; flexible wood; Poly(vinyl alcohol; Chitosan	Adhesion/anti-adhesion; flexibility;	Van der Waals force;	Nasal infection	[27]
Double-network	PAAm/SA/TA matrix, AgNPs, Chitosan/bovine serum-AuNPs	Water-responsive Janus adhesion, Adaptive antibacterial property, pH-responsive monitoring	Covalently cross-linking; Ionically cross-linking; Hydrogen bond	Diagnosis and treatment of catheters-associated infections	[28]
Interfacial polymerization at an air/solution interface	N-isopropyl acrylamide, Allylamine hydrochloride, Dopamine	Thermoresponsive; strong adhesion; on-demand adhesion	Cross-linking reaction	Underwater adhesives	[29]
Penetration cross-linking method	Oxidized sodium alginate, Carboxymethyl chitosan	Asymmetric structure; antibacterial;	Schiff base	Treatment of an infected extraction socket	[30]
Two-layer	Poly(vinyl) alcohol, Sodium alginate, Thioglycellulose chitosan, N, N’-bisacryloyl cysteamine, bFGF	Adhesion/anti-adhesion; Antibacterial; Precise release of drugs	Electrostatic bonding; Amide bonding; Disulfide bonding; Hydrogen bond	Tendon injury repair	[31]
Three-layer(layer-by-layer)	Sulfobetaine methacrylate; poly(vinyl) alcohol, tannic acid; polyacrylic acid, polyethyleneimine	Adhesion/anti-adhesion; anti-swelling	Hydrogen bond; Electrostatic interaction; Cation-Π interaction	Anti-postoperative adhesion; tissue adhesive	[32]
Release-Occupy strategy	Poly(vinyl) alcohol, Calcium alginate, Ionic liquids, Ethylene glycol	Adhesion/anti-adhesion; Conductivity; Mechanical and sensing stability	Hydrogen bond;	Wearable sensor	[33]
Unilateral graft modification	Methacrylated hyaluronate acid (HAMA); Poly sulfobetaine-type zwitterion (SBMA); Ketoglutarate (KA); Poly(acrylic acid-co- N-hydroxysuccinimide acrylate) [P(AA-co-AA-NHS)]	Adhesion/anti-adhesion;	Electrostatic interaction; Semi-interpenetrating	Tissue repair, Prevention of post-operative adhesions	[34]
Spin-coating process	Chitosan-gelatin/rhein-graphene oxide; poly(vinyl) alcohol (PVA)- poly(acrylic acid) (PAA))	Adhesion/anti-adhesion; Antibacterial activity	Phase separation; hydrogen-bond	Prevent postoperative tissue adhesion; Multidrug-resistant *Staphylococcus aureus*-infected open wound	[35]

**Table 2:** Advantages and disadvantages of various methods for preparing Janus hydrogel.

Preparation Method	Advantage	Disadvantage
Layer by layer	High interface accuracy; high flexibility of components	Processing time, limited number of layers; difficulty in achieving scale
One-pot	Simple operation; short time consumption; easy scalability.	Blurry interface; poor structural controllability
Self-assembly	No external force required; low energy consumption; capable of forming complex topologies	Uncontrollable dynamics; poor reproducibility
Electrospinning	It can be used to prepare fibrous Janus structures High specific surface area	Weak interface adhesion; poor solvent compatibility
Unilateral dipping	Equipment is simple. low cost	Asymmetry is incomplete, Thickness is uneven
External field	Real-time dynamic regulation; fast response speed (magnetic/electrical field/pH/temperature)	Require special equipment; limited for use inside the body
Cyclic freeze–thaw	Enhance mechanical strength; no need for chemical crosslinking agents	Long cycle; uncontrolled aperture
3D printing	Complex structure customization; multi-material integration; precise spatial distribution	Resolution limitation; The requirements for biological ink are extremely strict
Template strategy	Simple operation; high structural accuracy; compatible with hard/soft material composites	The template removal process is prone to leaving behind impurities; the removal of large-sized templates may lead to the collapse of the structure.
Unilateral graft modification	Chemical bonding interface; controllable thickness of the modified layer; gradient functionalization	Complex process; deep grafting efficiency decline; high-speed grafting may damage the substrate

## Data Availability

No new data were created or analyzed in this study.
